# Protective paraspeckle hyper-assembly downstream of TDP-43 loss of function in amyotrophic lateral sclerosis

**DOI:** 10.1186/s13024-018-0263-7

**Published:** 2018-06-01

**Authors:** Tatyana A. Shelkovnikova, Michail S. Kukharsky, Haiyan An, Pasquale Dimasi, Svetlana Alexeeva, Osman Shabir, Paul R. Heath, Vladimir L. Buchman

**Affiliations:** 1 School of Biosciences, Cardiff University, Museum Avenue, Cardiff, CF10 3AX UK; 2 Institute of Physiologically Active Compounds Russian Academy of Sciences, 1 Severniy proezd, Chernogolovka, Moscow Region, Russian Federation 142432; 3 The Sheffield Institute for Translational Neuroscience, 385A Glossop Road, Sheffield, S10 2HQ UK

**Keywords:** ALS, TDP-43, Paraspeckle, NEAT1

## Abstract

**Background:**

Paraspeckles are subnuclear bodies assembled on a long non-coding RNA (lncRNA) NEAT1. Their enhanced formation in spinal neurons of sporadic amyotrophic lateral sclerosis (ALS) patients has been reported but underlying mechanisms are unknown. The majority of ALS cases are characterized by TDP-43 proteinopathy. In current study we aimed to establish whether and how TDP-43 pathology may augment paraspeckle assembly.

**Methods:**

Paraspeckle formation in human samples was analysed by RNA-FISH and laser capture microdissection followed by qRT-PCR. Mechanistic studies were performed in stable cell lines, mouse primary neurons and human embryonic stem cell-derived neurons. Loss and gain of function for TDP-43 and other microRNA pathway factors were modelled by siRNA-mediated knockdown and protein overexpression.

**Results:**

We show that de novo paraspeckle assembly in spinal neurons and glial cells is a hallmark of both sporadic and familial ALS with TDP-43 pathology. Mechanistically, loss of TDP-43 but not its cytoplasmic accumulation or aggregation augments paraspeckle assembly in cultured cells. TDP-43 is a component of the microRNA machinery, and recently, paraspeckles have been shown to regulate pri-miRNA processing. Consistently, downregulation of core protein components of the miRNA pathway also promotes paraspeckle assembly. In addition, depletion of these proteins or TDP-43 results in accumulation of endogenous dsRNA and activation of type I interferon response which also stimulates paraspeckle formation. We demonstrate that human or mouse neurons in vitro lack paraspeckles, but a synthetic dsRNA is able to trigger their de novo formation. Finally, paraspeckles are protective in cells with compromised microRNA/dsRNA metabolism, and their assembly can be promoted by a small-molecule microRNA enhancer.

**Conclusions:**

Our study establishes possible mechanisms behind paraspeckle hyper-assembly in ALS and suggests their utility as therapeutic targets in ALS and other diseases with abnormal metabolism of microRNA and dsRNA.

**Electronic supplementary material:**

The online version of this article (10.1186/s13024-018-0263-7) contains supplementary material, which is available to authorized users.

## Background

Amyotrophic lateral sclerosis (ALS), the most common form of motor neuron disease, is a severe adult-onset neuromuscular disease affecting motor neurons in the spinal cord, brainstem and motor cortex. Up to 90% of ALS cases are sporadic (sALS), the rest 10% bear a strong genetic component (familial ALS, fALS), and currently mutations in more than 20 genes are known to cause fALS [1]. The complexity of the disease hinders development of ALS therapeutics, and those two drugs that have been approved for the treatment of ALS so far, riluzole and edaravone, have very limited efficacy.

A multifunctional RNA-binding protein TDP-43 encoded by *TARDBP* gene is believed to be the main culprit in ALS: TDP-43 pathology is typical for ~ 95% of sALS cases and for fALS cases caused by *C9ORF72* gene mutation [2]; in addition, dozens of mutations in *TARDBP* have been identified in fALS and sALS patients [3, 4]. Hallmarks of all these ALS cases include protein clearance from the nucleus, its cytoplasmic accumulation and aggregation [5, 6]. Therefore, both loss and gain of TDP-43 function are implicated in ALS however the relative contribution of these two mechanisms is still debated.

The paraspeckle is a prototypical nuclear body localized on the border of splicing speckles [7]. A long non-coding RNA (lncRNA) NEAT1 serves as a scaffold for paraspeckles, spatially organizing a variety of proteins by direct binding or piggy-back mechanism [8–11]. The *NEAT1* locus produces two transcripts, NEAT1_1 and NEAT1_2. The longer NEAT1 isoform, NEAT1_2, is essential for paraspeckle assembly [10, 12]. Functions of paraspeckles described so far include nuclear retention of specific RNAs, including inverted Alu repeat-containing transcripts; regulation of gene expression by sequestration of transcription factors; and modulation of miRNA biogenesis [13–16].

There is an established association of paraspeckles and their components with a variety of pathological states and conditions, from cancer to neurodegeneration. Paraspeckles protect cancer cells against DNA damage and replication stress, regulate hormone receptor signaling and hypoxia-associated pathways thereby increasing their survival [17–19]. Paraspeckles become enlarged in cells primed by viral or synthetic double-stranded (ds) RNAs and play an important role in antiviral response [14]. An unusually tight association of paraspeckle components with neurodegenerative conditions, and ALS in particular, has recently emerged. Firstly, enhanced paraspeckle formation has been reported in spinal motor neurons of sALS patients [20]. This finding was surprising because levels of the longer NEAT1 isoform, NEAT1_2, essential for paraspeckle formation, are very low in the adult nervous system [21]. Secondly, at least seven paraspeckle proteins, including TDP-43 and FUS, are genetically linked to ALS and a related condition, frontotemporal lobar degeneration (FTLD) [22–25]. FUS, a protein structurally and functionally similar to TDP-43, is required to build paraspeckles [8, 23]. TDP-43 association with paraspeckles has also been reported [8]. TDP-43 directly binds NEAT1, and this interaction is increased in the brain of FTLD patients [26, 27]. Overall, currently available data support the role of paraspeckles in molecular pathology of ALS, however the underlying mechanisms of their enhanced formation in spinal neurons are not understood.

In current study we show that loss of TDP-43 is sufficient to stimulate paraspeckle formation – a phenomenon likely linked to the function of TDP-43 in microRNA (miRNA) processing and as an RNA chaperone. Furthermore, we provide evidence that paraspeckles are protective in cells with impaired function of the miRNA machinery and those with activated dsRNA response. Finally, we show that enoxacin, an enhancer of the miRNA pathway, promotes paraspeckle formation.

## Methods

### Stable cell line maintenance, transfection and treatments

SH-SY5Y neuroblastoma cells and MCF7 cells were maintained in 1:1 mixture of Dulbecco’s Modified Eagle’s Medium and F12 medium supplemented with 10% fetal bovine serum (FBS), penicillin-streptomycin and glutamine (all Gibco, Invitrogen). For differentiation into neuron-like cells, SH-SY5Y cells were grown on poly-L-lysine (Sigma) coated coverslips in advanced DMEM/F12 (ADF)/Neurobasal A mixture supplemented with 10 μM all-trans retinoic acid (Sigma), B27 (Life Technologies) and BDNF (Miltenyi, 10 ng/ml) for 6 days. The following gene-specific siRNAs were used: ADAR1; Dicer; Drosha; FUS; Ago2; IFNB1 (all Life Technologies, Silencer®); TARDBP (Silencer Select®, s23829 and EHU109221, Mission® esiRNA, Sigma); NEAT1 (Silencer Select®, n272456). Scrambled negative control was AllStars from Qiagen. Plasmids for expression of TDP-43 dNLS and TDP-43 C-termical fragment are described elsewhere [28]. Cells were transfected with siRNA (400 ng/well), plasmid DNA (200 ng/well) or poly(I:C) (Sigma, 250 ng/well) using Lipofectamine2000 (Life Technologies) in 24-well plates. TDP-43 specific shRNA plasmid was from Sigma (MISSION® SHCLNG-NM_007375). To delete the NLS of endogenous TDP-43, Feng Zhang lab’s Target Finder (http://crispr.mit.edu/) was used to identify guide RNA target sequences flanking the genomic region of *TARDBP* gene encoding NLS. Respective forward and reverse oligonucleotides for two pairs of guides were annealed and cloned into pX330-U6-Chimeric_BB-CBh-hSpCas9 (pX330) vector provided by Feng Zhang (Addgene deposited plasmid) as described [29]. MCF7 cells were transfected with plasmids encoding upstream and downstream guide RNAs (500 ng/well) using Lipofectamine2000 and analysed after 72 h. Guide RNA sequences: T1: 5’-TTATTTAGATAACAAAAGAAAAA-3′, T2: 5’-AACATCCGATTTAATAGTGT-3′, T3: 5’-GGAATTCTGCATGCCCCAGATGC-3′, T4: 5’-ACATCCGATTTAATAGTGTT-3′. Cellular treatments were as follows: 1 × 10^4^ IU interferon beta-1a (IFNbeta), 0.5 μg/ml LPS, 100 μg/ml zymosan, 50 μM suramin, 500 nM TSA, 2 mM sodium butyrate, 10 and 50 μM enoxacin, 10 μM riluzole, 10 μM edaravone (all Sigma). Human ES cell derived neurons were transfected with 15 μg/well of poly(I:C) using FuGENE®HD (Promega). Enoxacin, edaravone and riluzole toxicity was assessed using resazurin-based CellTiter-Blue Cell Viability Assay (Promega).

### Primary culture of mouse neurons

Primary cultures of mouse hippocampal neurons were prepared from P0 CD1 mice as described [28] and maintained for 5–14 days.

### Differentiation of human ES cells into motor neuron enriched cultures

Cultures of human neural precursor cells (NPCs) and motor neurons differentiated from H9 hES cell line were prepared as described previously [30]. Briefly, hES cells were maintained in mTESR2 media (Stemcell Technologies) on Matrigel® (Corning) coated dishes. Confluent hES H9 cultures were switched to differentiation medium composed of ADF supplemented with SB431542 (10 μM, Abcam). Purmorphamine (1 μM, Cayman Chemicals) and retinoic acid (0.1 μM, Sigma) were added on Day 4. On Day 8, cells were split in 1:2 ratio and on Day 16, NPCs were dissociated using Accutase®, plated onto Matrigel® coated dishes and cultured in ADF with GlutaMAX, penicillin-streptomycin, B27 (12587–010) and N2 supplements (all Life Technologies) and BDNF (Miltenyi, 10 ng/ml). On Day 23, Accutase® was used to re-plate neurons on dishes/coverslips at desired density. Neurons were cultured in 50:50 mixture of ADF/Neurobasal A with the above supplements until Day 40.

### Immunocytochemistry and RNA-FISH on cultured cells

Cells were fixed on coverslips with 4% paraformaldehyde on ice for 15 min and permeabilized in cold methanol (or 70% ethanol in case of RNA-FISH). Coverslips were incubated with primary antibodies diluted in blocking solution (5% goat serum in 0.1% Triton X-100/PBS) for 1 h at RT or at 4 °C overnight. Secondary Alexa488- or Alexa546-conjugated antibody was added for 1 h at RT. For RNA-FISH, commercially available NEAT1 and MALAT1 probes (Stellaris® FISH Probes against human NEAT1, middle segment or 5′ segment, or human MALAT1, all Biosearch Technologies) were used as per standard protocol. Fluorescent images were taken using BX61 microscope equipped with F-View II camera and processed using CellF software (all Olympus). Paraspeckle quantification (number of individual paraspeckles per DAPI-visualised nucleus) was performed manually, by the same person for all conditions, blinded to the experimental condition. Clusters of paraspeckles were counted as a single paraspeckle. For quantification of cleaved caspase 3 positive cells, ‘Analyze particles’ tool of Image J software was used (8–10 fields were analysed per condition).

### RNA analysis

Total cellular RNA was extracted using GenElute total RNA kit (Sigma) and possible DNA contamination was removed using RNase free DNase kit (Qiagen). First-strand cDNA synthesis were performed using random primers and Superscript IV (Invitrogen). For analysis of miRNA levels, RNA was extracted with QIAzol (Qiagen) followed by reverse transcription with Qiagen miScript II RT Kit. Real-time qPCR was conducted using SYBR green master mix as described [28]. For miRNA quantification, forward miRNA-specific primers were used in combination with the universal reverse primer (unimiR). All primer sequences are given in Table 1.

**Table 1:** Primers used in the study

Target	Forward	Reverse
GAPDH	5’-TCGCCAGCCGAGCCA-3′	5’-GAGTTAAAAGCAGCCCTGGTG − 3′
NEAT1 total	5’-CTCACAGGCAGGGGAAATGT-3′	5’-AACACCCACACCCCAAACAA-3′
NEAT1_2	5’-AGAGGCTCAGAGAGGACTGTAACCTG-3′	5′-TGTGTGTGTAAAAGAGAGAAGTTGTGG-3’
TDP-43	5’-TCAGGGCCTTTGCCTTTGTT-3’	5’-TGCTTAGGTTCGGCATTGGAT-3’
IL8	5’-ACACTGCGCCAACACAGAAA-3′	5’-CCTCTGCACCCAGTTTTCCT-3’
ADARB2	ATATTCGTGCGGTTAAAAGAAGGTG	ATCTCGTAGGGAGAGTGGAGTCTTG
Alu RNA	5’-GAGGCTGAGGCAGGAGAATCG-3’	5’-GTCGCCCAGGCTGGAGTG-3’
DICER	5’-TTAACCTTTTGGTGTTTGATGAGTGT-3’	5’-GCGAGGACATGATGGACAATT-3’
DROSHA	5’-CGGCCCGAGAGCCTTTTAT-3’	5’-TGCACACGTCTAACTCTTCCA-3’
ADAR1	5’-TTGTCAACCACCCCAAGGT-3’	5’-CCATCAGCCAGACACCAGTT-3’
AGO2	5’-CACCATGTACTCGGGAGCC-3’	5’-TCCCAAAGTCGGGTCTAGGT-3’
FUS	5’-GCGGGGCTGCTCAGT-3’	5’-TTGGGTTGCTTGTTGGGTAT-3’
CHOP	5’-TTAAAGATGAGCGGGTGGC-3′	5’-GCTTTCAGGTGTGGTGATGTA-3’
CXCL10	5’-TGCCATTCTGATTTGCTGCC-3’	5’-ATGCTGATGCAGGTACAGCG-3’
IFNB1	5’-ACGCCGCATTGACCATCTAT-3’	5’-AGCCAGGAGGTTCTCAACAA-3’
IFNA1	5’-TCTGCTATGACCATGACACGAT-3’	5’-CAGCATGGTCCTCTGTAAGGG-3’
IFNA2	5’-AGGAGGAAGGAATAACATCTGGTC-3’	5’-GCAGGGGTGAGAGTCTTTGAA-3’
MALAT1	5’-GGATCCTAGACCAGCATGCC-3’	5′- AAAGGTTACCATAAGTAAGTTCCAGAAAA-3’
IFIH1	5’-GCATGGAGGAGGAACTGTTGA-3’	5’-GCATGGAGGAGGAACTGTTGA-3’
CYCS	5’-TCGTTGTGCCAGCGACTAAA-3’	5’-GCTTGCCTCCCTTTTCAACG-3’
STAT1	5’-CTGTGCGTAGCTGCTCCTTT-3’	5’-GGTGAACCTGCTCCAGGAAT-3’
MYD88	5’-TGACCCCCTGGGGCAT-3’	5’-AGTTGCCGGATCATCTCCTG-3’
Pri-miR-17–92	5’-CAGTAAAGGTAAGGAGAGCTC AATCTG-3’	5’-CATACAACCACTAAGCTAAAGAAT AATCTGA-3’
Pri-miR-15a	5’-CCTTGGAGTAAAGTAGCAGCAC-3’	5’-CCTTGTATTTTTGAGGCAGCAC-3’
miR-18a	5’-CATCATCGGTAAGGTGCATC-3’	5’-GAATCGAGCACCAGTTACGC-3′ (unimiR)
miR-92a	5’-GAGTCTATTGCACTTGTCCC-3’	unimiR
miR-106a	5’-AAAAGTGCTTACAGTGCAGGTAG-3’	unimiR

### RNA immunoprecipitation (RIP) and PCR analysis

MCF7 cells were transfected with equal amounts of plasmids to express GFP-tagged FUS or NONO together with TARDBP siRNA or scrambled control siRNA. After 48 h, cells were scraped in RIP buffer prepared using RNase-free water (1xPBS with 1% Triton-X100 and protease inhibitors cocktail). Cells were left on ice for 10 min with periodic vortexing, and the lysate was centrifuged at 13,000 rpm for 10 min. GFP-Trap® beads (Chromotek) were washed in RIP buffer 4 times and added directly to cleared cell lysates with subsequent rotation at + 4 °C for 3 h. Beads were washed 4 times in RIP buffer and RNA was eluted by resuspension in TRI-reagent (Sigma). RNA was purified according to manufacturer’s protocol, and equal amounts of RNA were used for cDNA synthesis as described above.

### Protein analysis

Total cell lysates were prepared for Western blot by lysing cells in wells in 2× Laemmli (loading) buffer followed by denaturation at 100 °C for 5 min. Proteins were resolved by SDS-PAGE and transferred to PVDF membrane (Amersham) by semi-dry transfer. The membrane was blocked in 4% non-fat milk in TBST and incubated in primary antibodies prepared in milk or 5% BSA overnight. Secondary HRP-conjugated antibodies were from Amersham. For detection of proteins, WesternBright Sirius ECL reagent (Advansta) was used. β-actin was used for normalisation.

### Primary antibodies

The following commercial primary antibodies were used: TDP-43 (rabbit polyclonal, 10782–2-AP, Proteintech and mouse monoclonal, MAB7778-SP, R&D Biosystems); FUS (rabbit polyclonal, Proteintech, 11570–1-AP); p54nrb/NONO (rabbit polyclonal C-terminal, Sigma); PSF/SFPQ (rabbit monoclonal, ab177149, Abcam); Tuj (β-Tubulin III, mouse monoclonal, Sigma); dsRNA (mouse monoclonal, J2, Kerafast); cleaved caspase 3 (rabbit polyclonal, 9661, Cell Signaling); NF-κB p65 (rabbit monoclonal, D14E12, Cell Signaling); IFIT3 (rabbit polyclonal, Bethyl); p-eIF2α (rabbit monoclonal, ab32157, Abcam); p-PKR (rabbit polyclonal, Thr451, ThermoFisher); PKR (mouse monoclonal, MAB1980-SP, R&D Systems); eIF2α (rabbit monoclonal, D7D3, Cell Signaling); β-actin (mouse monoclonal, A5441, Sigma). Antibodies were used at 1:500–1:1000 dilution for all applications.

### Analysis of human tissue samples

Human spinal cord paraffin sections from a panel of clinically and histopathologically characterised ALS cases and neurologically healthy individuals were obtained from the Sheffield Brain Tissue Bank and MRC London Neurodegenerative Diseases Brain Bank (Institute of Psychiatry, King’s College London). Consent was obtained from all subjects for autopsy, histopathological assessment and research were performed in accordance with local and national Ethics Committee approved donation. Human spinal cord sections were 7 μm thick. For conventional RNA-FISH, slides were boiled in citrate buffer for 10 min, washed in 2xSSC prepared with DEPC-treated water and incubated with NEAT1 probe (Stellaris® FISH Probes against human NEAT1 5′ segment, Biosearch Technologies) diluted in hybridisation buffer (10% formamide/2xSSC, 5 μl probe in 200 μl buffer per slide under a 24 × 60 mm coverslip) in a humidified chamber at 37 °C overnight. Nuclei were co-stained with DAPI. Paraspeckles were analysed using BX61 microscope/F-View II camera (Olympus) at 100× magnification. For RNAscope® ISH analysis, Hs-NEAT1-long (411541) probe (Advanced Cell Diagnostics) was used according to manufacturer’s instructions. For qRT-PCR analysis, total RNA was extracted from thick frozen spinal cord sections and cDNA prepared using Ready-To-Go You-Prime First-Strand Beads (GE Healthcare). For laser capture microdissection (LCM), frozen spinal cord sections (total of 5 sections per patient/case) were cut into 5–10 μm thin sections using a cryostat, mounted on glass slides and fixed in cold acetone for 3 min. Sections were stained using toluidine blue, dehydrated in ascending alcohol series for 30 s and placed in xylene for 1 min. The PixCell® II Microdissection system (Applied Biosystems) was used for LCM. Motor neurons from the anterior grey horn were laser captured (300–500 or 30–80 per patient for healthy controls and ALS cases respectively), with the Macro-LCM cap films peeled and placed in test tubes with 50 μl extraction buffer (Pico Pure® RNA Isolation Kit; Thermo Fisher Scientific) on ice. The extracted films were incubated at 42 °C with the extraction buffer for 30 min and frozen at -80 °C until RNA extraction. Total RNA purification was performed using the above kit as per manufacturer’s instructions. RNA samples were analysed using the Agilent RNA 6000 Pico Kit (Agilent Technologies®) and used for qRT-PCR.

### Statististical analysis

GraphPad Prism software was used for statistical analysis. Statistical test used in each case is indicated in the figure legend. N indicates the number of biological replicates. On all graphs, error bars represent SEM.

## Results

### Presence of paraspeckles in the spinal cord neurons is a hallmark of sALS and fALS

**Fig. 1:**
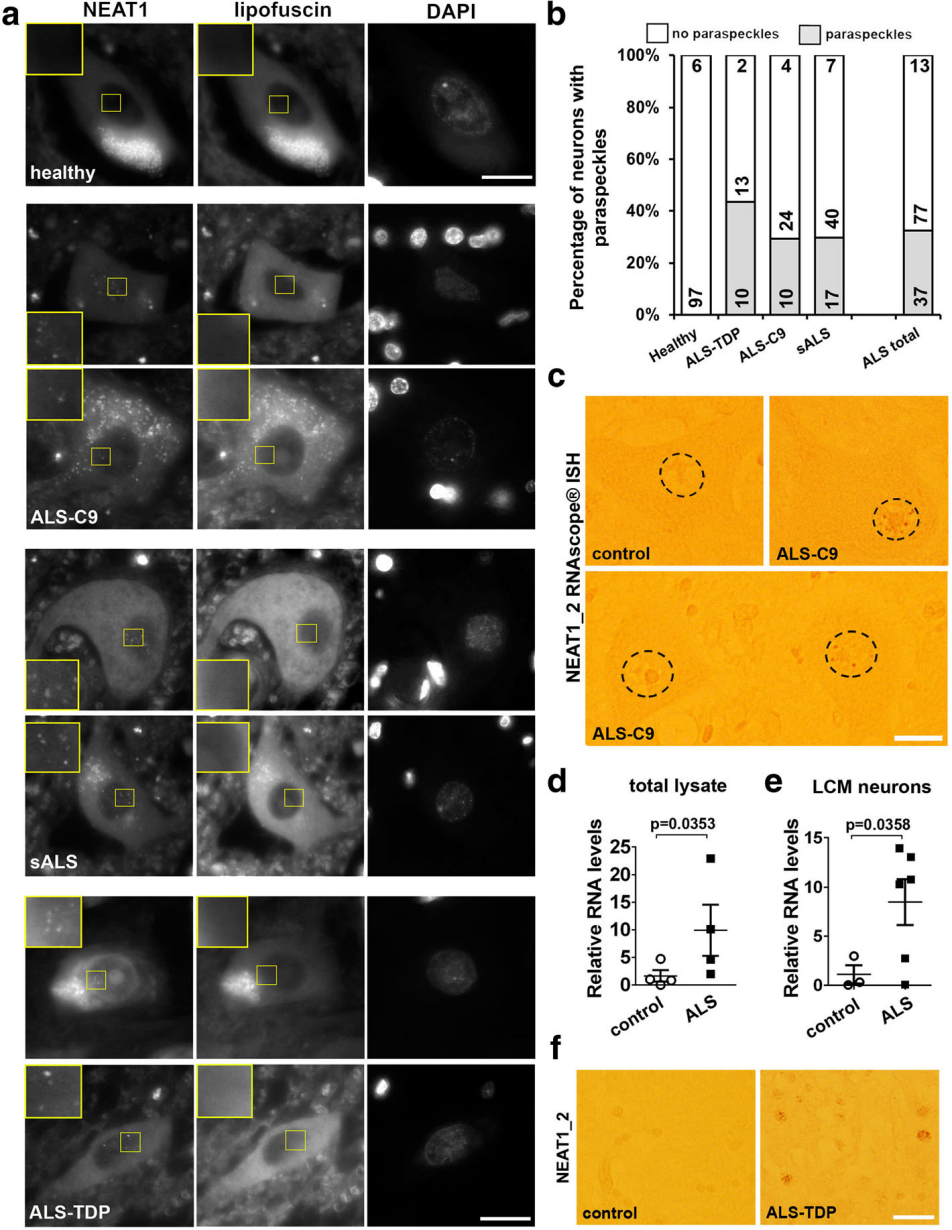
Paraspeckles are formed in the spinal cord of sALS and fALS patients but not healthy controls. **a** and **b** Examples of spinal motor neurons with paraspeckles (**a**) and their quantification (**b**) in ALS patients with different disease aetiology. Paraspeckles were visualised in the spinal cord sections of a cohort of fALS and sALS patients as well as neurologically normal control individuals using RNA-FISH with a fluorescent (Quasar 570) probe mapping to the 5′ portion of NEAT1. Images were also taken in the FITC channel to distinguish between specific NEAT1 signal and green autofluorescence from lipofuscin (**a**). The fraction of neurons with identifiable paraspeckles in the spinal anterior horn of aetiologically different ALS cases and control individuals was quantified and plotted separately for fALS with *TARDBP* mutations (ALS-TDP), fALS with *C9ORF72* repeat expansion (ALS-C9) and sALS cases (**b**). The top figure within each bar corresponds to the number of cases analysed and the figures below - to the number of individual neurons negative or positive for the presence of paraspeckles. Scale bars, 10 μm. **c** Examples of paraspeckle-containing neurons in the ALS spinal cord visualised with RNAscope® NEAT1_2 specific probe. In the bottom panel, a paraspeckle-positive (right) and a paraspeckle-negative (left) neurons, found adjacent to each other, are shown. Nuclei are circled. Scale bar, 10 μm. **d** NEAT1 levels in the total RNA samples extracted from transversely cut spinal cord blocks of ALS patients and healthy controls analysed by qRT-PCR (*n* = 4 for control and ALS patients, including two sALS and two ALS-C9 cases, Mann-Whitney *U*-test). **e** NEAT1_2 levels in neurons microdissected from the spinal anterior horn of ALS patients and healthy controls analysed by qRT-PCR (*n* = 3 for control and *n* = 6 for ALS cases, including three sALS and three ALS-C9 cases, Mann-Whitney *U*-test). **f** Paraspeckles in glial cells in the ALS spinal cord visualised with RNAscope® ISH using NEAT1_2 specific probe. Representative images of the spinal cord for a control individual and an ALS patient are shown. Scale bar, 20 μm

Augmented paraspeckle assembly has been previously reported in sALS spinal cord neurons as compared to non-ALS controls [20]. We sought to verify this result in a separate cohort of sALS cases as well as to extend this analysis to fALS. In total, 7 sALS cases, 2 cases with *TARDBP* mutations and 4 cases with *C9ORF72* mutations alongside with 6 healthy controls were examined by RNA-FISH with NEAT1 probe. No neurons with paraspeckles were detected in the spinal cord of healthy individuals (97 neurons analysed), however such neurons were present in up to 40% of neurons in all ALS cases examined (Fig. 1a and b). We also confirmed the presence of paraspeckles in ALS motor neurons using RNAscope® ISH (Fig. 1c). Consistently, qRT-PCR analysis of spinal cord tissue from four healthy controls and four ALS patients demonstrated elevated NEAT1 levels in the latter group (Fig. 1d). We further performed laser capture microdissection (LCM) of spinal neurons in the ventral horn and analysed NEAT1_2 levels by qRT-PCR (*n* = 3 for controls and *n* = 6 for ALS patients, including three sALS and three ALS-C9 cases). NEAT1_2 levels were indeed significantly upregulated in LCM neurons of ALS patients (Fig. 1e). Finally, using RNAscope® ISH, we also analysed the presence of paraspeckles in non-neuronal cells. Wide-spread paraspeckle assembly in glial cells in ALS spinal cord was observed (n = 6 for controls and *n* = 4 for ALS patients, including two sALS, one ALS-TDP and one ALS-C9 case) (Fig. 1f).

Thus, de novo paraspeckle formation is typical for spinal motor neurons and glial cells of individuals affected by ALS with primary or secondary TDP-43 pathology.

### Loss of TDP-43 but not its cytoplasmic accumulation or aggregation results in paraspeckle hyper-assembly

We next sought to determine possible mechanisms underlying paraspeckle hyper-assembly in ALS. TDP-43 pathology in the spinal cord is very common in ALS, being present in almost all sALS cases, fALS cases caused by mutations in *TARDBP* gene itself as well as those caused by *C9ORF72* gene repeat expansions [2–4, 31]. TDP-43 has been identified as a paraspeckle protein [20], thus we tested the possibility that TDP-43 dysfunction affects paraspeckle assembly.

**Fig. 2:**
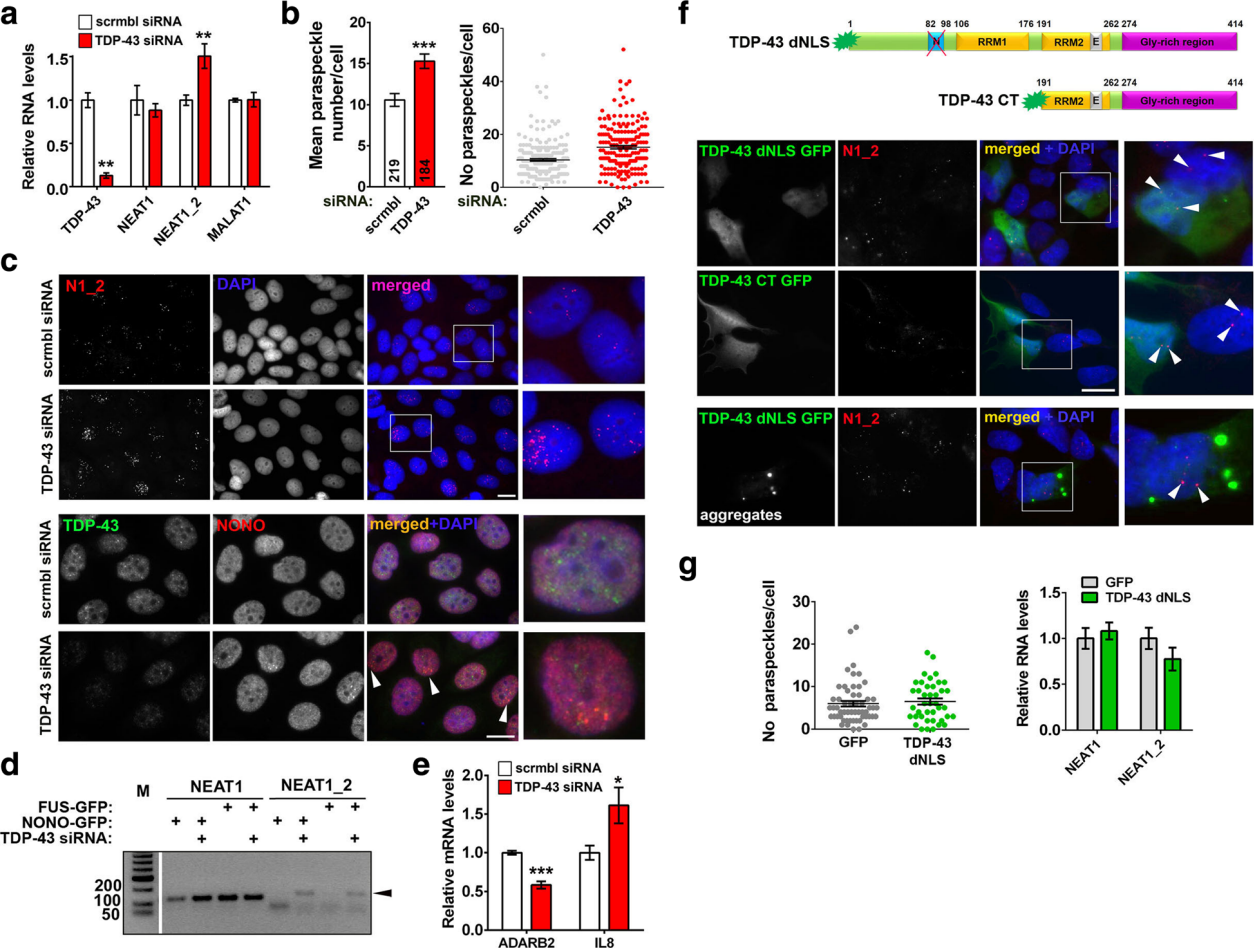
TDP-43 depletion but not its cytoplasmic accumulation or aggregation stimulates paraspeckle assembly in stable cell lines. **a** TDP-43 siRNA-mediated knockdown upregulates NEAT1_2. MCF7 cells were transfected with scrambled or TDP-43 siRNA and analysed 48 h post-transfection by qRT-PCR (n = 6). **p < 0.01 (Mann-Whitney *U*-test). **b** and **c** TDP-43 depletion auguments paraspeckle assembly in MCF7 cells. Quantification (**b**) and representative images (**c**) are shown. RNA-FISH with NEAT1_2 probe (**c**, top panels) or anti-NONO staining (**c**, bottom panels) were used to visualise paraspeckles; arrowheads indicate clusters of paraspeckes. The number of cells analysed is indicated in the bottom of each bar (**b**) (***p < 0.0001, Student’s *t*-test). **d** TDP-43 depletion enhances interaction of NEAT1_2 with core paraspeckle proteins NONO and FUS. GFP-tagged NONO or FUS was co-transfected into MCF7 cells together with scrambled siRNA or TDP-43 siRNA. NEAT1_2 and total NEAT1 were detected in GFP pull-down samples by RT-PCR. Arrowhead indicates the specific band for NEAT1_2 primer pair. **e** Expression of paraspeckle-regulated genes in MCF7 cells depleted of TDP-43 as measured by qRT-PCR (n = 6 or 8). *p < 0.05, ***p < 0.001 (Mann-Whitney *U*-test). **f** and **g** Expression of TDP-43 lacking nuclear localisation signal (TDP-43 dNLS) or TDP-43 C-terminal 25 kDa fragment (TDP-43 CT) does not affect paraspeckles or NEAT1 levels. Representative images of paraspeckles in transfected SH-SY5Y cells (**f**) and their quantiation (**g**, *n* = 56 and *n* = 41 for GFP- and TDP-43 dNLS-expressing cells respectively) are shown. Scale bars are 10 μm in all panels

Hallmarks of TDP-43 proteinopathy are clearance of the protein from the nucleus and its accumulation and aggregation in the cytoplasm [5, 6]. We first modelled loss of TDP-43 function in two stable cell lines. By using specific siRNA, ~ 90 and 50% TDP-43 knockdown was achieved in MCF7 and neuroblastoma SH-SY5Y cells, respectively (Fig. 2a; Additional file 1: Fig. S1a). TDP-43 depletion led to a significant increase of the number of paraspeckles per nucleus (Fig. 2b and c; Additional file 1: Fig. S1b). Consistently, the paraspeckle-specific NEAT1 isoform, NEAT1_2, was upregulated in cells transfected with TDP-43 siRNA (Fig. 2a; Additional file 1: Fig. S1a). Similar results were obtained using an independent TDP-43 siRNA pool and an shRNA targeting TDP-43 (Additional file 1: Fig. S1c and d). In contrast, we did not observe changes in the levels or distribution of another abundant lncRNA, MALAT1, a component of splicing speckles (Fig. 2a; Additional file 1: Fig. S1e). Levels of core paraspeckle proteins SFPQ, NONO and FUS were also unaffected by TDP-43 knockdown (Additional file 1: Fig. S1f). We next examined whether TDP-43 knockdown would result in enhanced association of core paraspeckle proteins with NEAT1_2. Plasmids to overexpress GFP-tagged FUS or NONO proteins were co-transfected with scrambled or TDP-43 siRNA followed by RNA immunoprecipitation with GFP-Trap beads. Indeed, by PCR, both FUS and NONO demonstrated increased association with NEAT1_2 in TDP-43 depleted cells (Fig. 2d). To verify that paraspeckles formed in TDP-43 depleted cells are functional, we measured the expression of established paraspeckle-dependent genes, *IL8* and *ADARB2,* known to be positively and negatively regulated by paraspeckles, respectively [13, 14]. Indeed, IL8 mRNA was upregulated and ADARB2 mRNA decreased upon TDP-43 knockdown (Fig. 2e).

Since nuclear clearance of TDP-43 in ALS is coupled to its cytoplasmic accumulation and aggregation, we next evaluated the effect of cytoplasmic TDP-43 on paraspeckles. TDP-43 lacking nuclear localization signal (TDP-43 dNLS) and a C-terminal TDP-43 fragment corresponding to ~ 25 kDa TDP-43 cleavage product (TDP-43 CT, aa. 191–414), both characterized by predominantly cytoplasmic distribution, were transiently expressed in neuroblastoma cells (Fig. 2f). However, neither paraspeckle numbers nor NEAT1 levels were affected by these cytoplasmic proteins (Fig. 2f and g). TDP-43 dNLS forms cytoplasmic aggregates in a fraction of cells, but their presence also did not affect paraspeckles (Fig. 2g, bottom panel). Finally, in order to recapitulate simultaneous nuclear depletion and cytoplasmic accumulation of TDP-43, we targeted endogenous TDP-43 out of the nucleus while preserving its total cellular levels by CRISPR/Cas9 editing of the endogenous *TARDBP* gene. Cells were transiently transfected with plasmids for expression of two independent guide RNA pairs targeting upstream and downstream sequences encoding NLS of TDP-43 (Additional file 1: Fig. S1 g). For both guide RNA pairs tested, 15–20% transfected cells displayed partial TDP-43 redistribution to the cytoplasm which nevertheless did not enhance paraspeckle assembly (Additional file 1: Fig. S1 h), suggesting that substantial loss of nuclear TDP-43 is required to produce an effect on paraspeckles.

Overall, a decrease in cellular TDP-43 levels results in paraspeckle hyper-assembly.

### Compromising miRNA pathway results in enhanced paraspeckle assembly

**Fig. 3:**
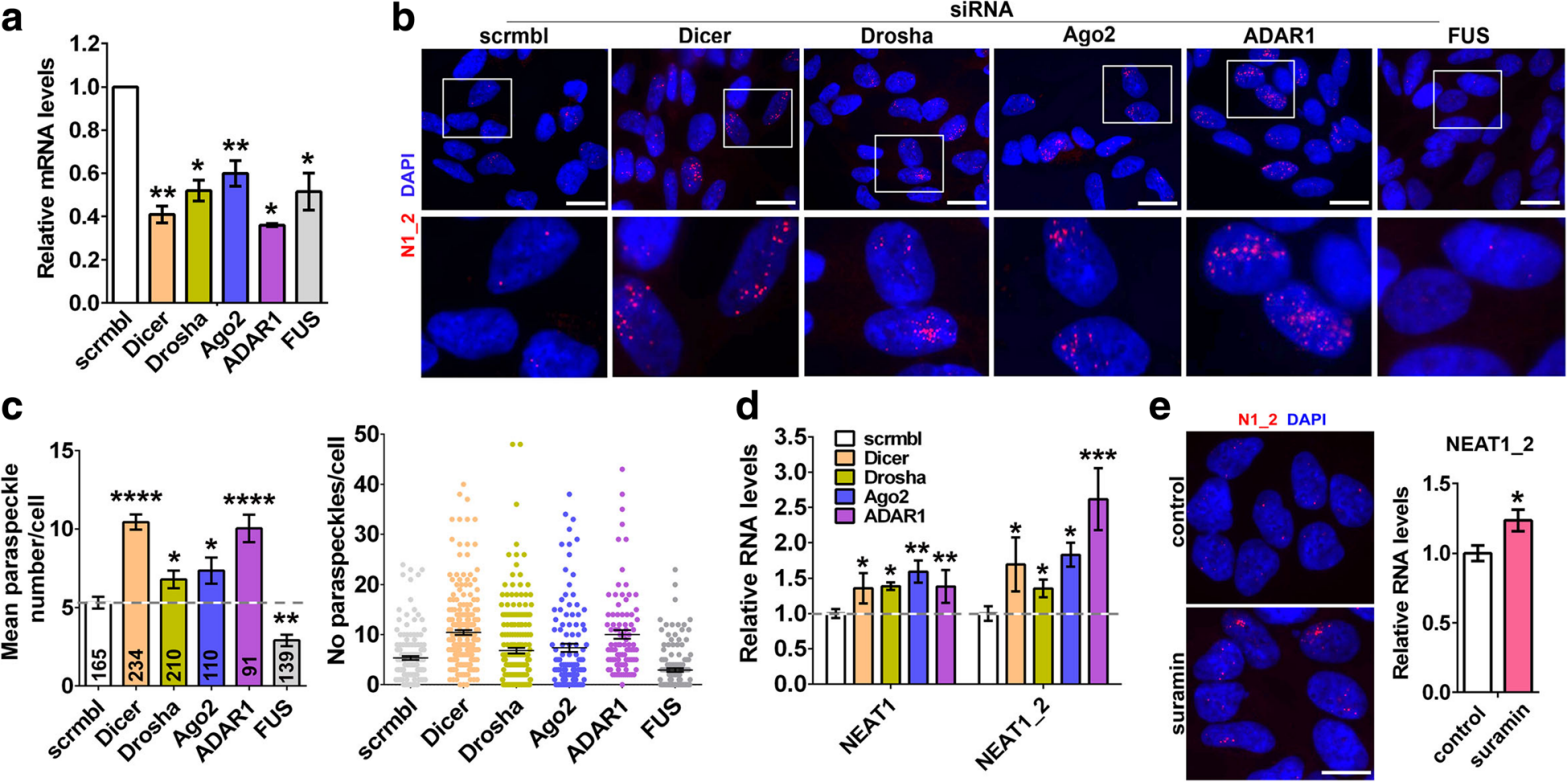
Enhanced paraspeckle assembly in cells with compromised function of the miRNA pathway. **a** Downregulation of Drosha, Dicer, Ago2, ADAR1 and FUS in neuroblastoma cells after transfection of specific siRNA as analysed by qRT-PCR (n = 4–6). *p < 0.05, **p < 0.01 (Mann-Whitney *U*-test). **b** and **c** Knockdown of Drosha, Dicer, Ago2 or ADAR1 results in increased paraspeckle formation. Representative images of cells (**b**) and paraspeckle quantification (**c**) are shown. In **c**, the mean number of paraspeckles per cell and frequencies of such cells were plotted; the number of cells analysed is indicated at the bottom of each bar. *p < 0.05, **p < 0.01; ****p < 0.0001 (one-way ANOVA with Holm-Sidak correction for multiple comparisons). **d** NEAT1 is upregulated in cells after knockdown of Drosha, Dicer, Ago2 and ADAR1 (n = 4–6). *p < 0.05, **p < 0.01, ***p < 0.001 (one-way ANOVA with Holm-Sidak correction for multiple comparisons). **e** Suramin stimulates NEAT1_2 expression and paraspeckle formation. Cells were treated with suramin for 24 h before collection for RNA-FISH and qRT-PCR analysis (n = 4). *p < 0.05 (Mann-Whitney *U*-test). In **a-d**, cells were analysed 48 post-transfection. Scale bars are 10 μm in all panels

TDP-43 is known to contribute to miRNA biogenesis at two different levels, enhancing the activity of the Microprocessor in the nucleus and of the Dicer complex in the cytoplasm [32, 33]. Recently, paraspeckles have been shown to contribute to pri-miRNA processing by spatially organizing the Microprocessor and enhancing its processivity [16]. We hypothesised that augmented paraspeckle assembly in cells depleted of TDP-43 might be a compensatory mechanism to counterbalance the effect of TDP-43 loss of function on miRNA processing. If this indeed is true, paraspeckle assembly should be also increased in cells with compromised function of the miRNA pathway. To test this hypothesis, we knocked down three core enzymes of the miRNA pathway, a Microprocessor component Drosha, the pre-miRNA processing ribonuclease Dicer and the RISC endonuclease Ago2, in neuroblastoma cells. In our analysis we also included ADAR1 protein recently reported to promote miRNA processing [34]. Using specific siRNAs, we achieved at least 40% knockdown for each of these genes (Fig. 3a). Consistent with the major role of Drosha in pri-miRNA processing, its knockdown led to the build-up of pri-miRNAs; both Drosha and TDP-43 knockdown also resulted in significantly diminished levels of select mature miRNAs (Additional file 2: Fig. S2).

RNA-FISH and paraspeckle quantification showed that downregulation each of the above proteins is accompanied by enhanced paraspeckle assembly (Fig. 3b and c) and upregulation of total NEAT1 and NEAT1_2 (Fig. 3d). Another ALS-linked protein, FUS, is structurally and functionally similar to TDP-43 and also plays a role in miRNA biogenesis [35], but it is a core paraspeckle protein required for paraspeckle integrity [8, 23]. As expected from its essential paraspeckle function, FUS depletion resulted in decreased paraspeckle numbers (Fig. 3b and c). Finally, a small molecule inhibitor of RISC loading, suramin [36], was also able to increase NEAT1_2 levels and promote paraspeckle assembly (Fig. 3e).

Thus, interfering with the function of the miRNA pathway causes NEAT1 upregulation and enhanced paraspeckle formation in cultured cells. This may represent one of the mechanisms behind the effect of TDP-43 loss of function on paraspeckles.

### Type I IFN signaling is activated in cells depleted of TDP-43 or other miRNA factors and stimulates paraspeckle formation

TDP-43 is known to bind and regulate long transcripts [37], and its loss correlates with accumulation of transcripts prone to form double stranded (ds) RNA [38]. Conspicuously, regulation of cellular response to viral dsRNA is one of the best characterized functions of paraspeckles [14, 39]. Therefore, we considered abnormal accumulation of endogenous dsRNA in TDP-43 depleted cells as another mechanism underlying the effect of TDP-43 loss of function on paraspeckles. Critically, miRNA pathway itself is the biggest cellular source of dsRNA, and its factors Dicer, Drosha and ADAR1 are known to limit the accumulation of transcripts with extensive secondary structure [40–42].

**Fig. 4:**
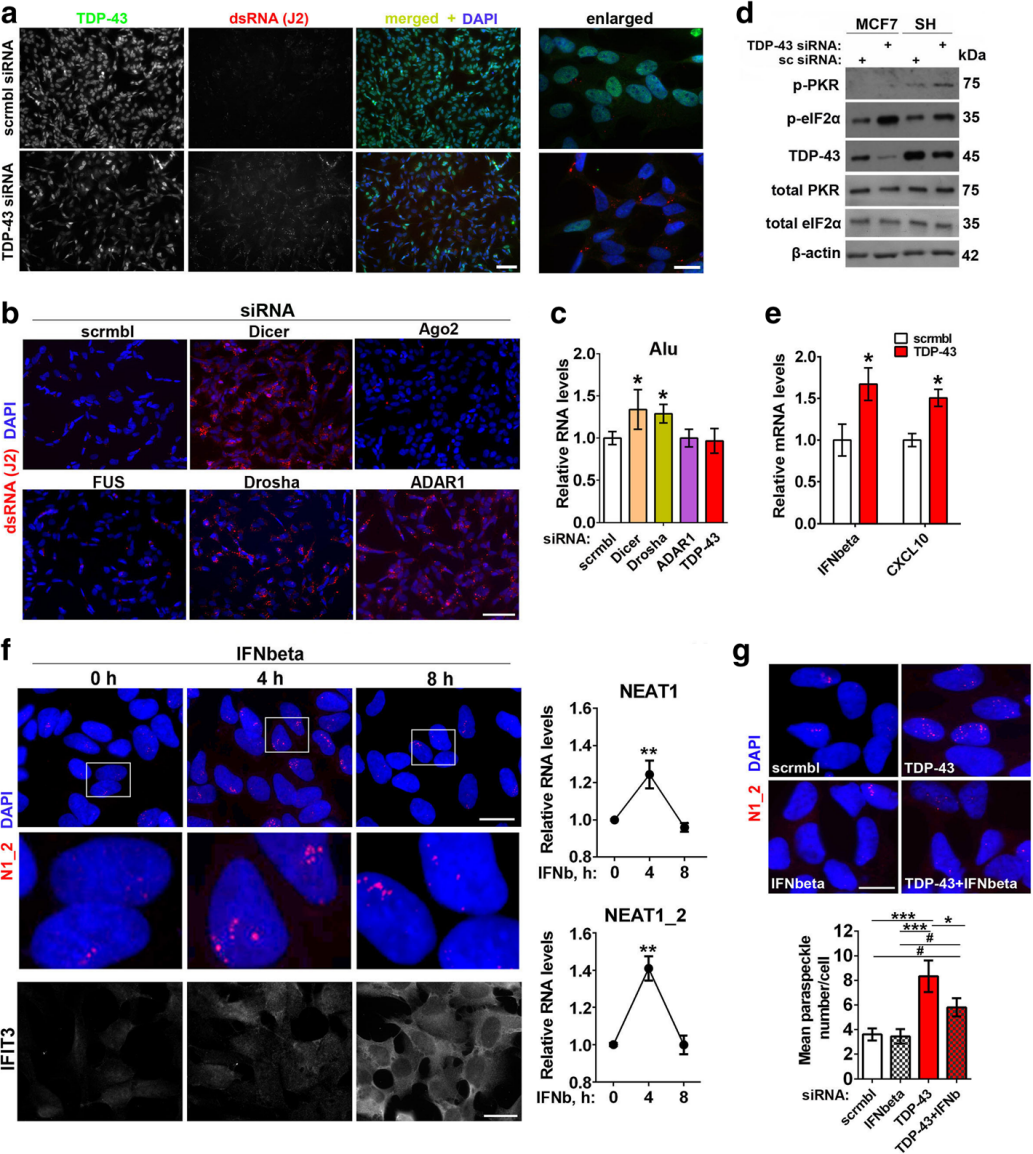
Endogenous dsRNA response and type I interferon promote paraspeckle hyper-assembly in stable cell lines. **a** and **b** Depletion of TDP-43, Dicer, Drosha, ADAR1 but not Ago2 or FUS causes intracellular build-up of dsRNA. dsRNA was detected by immunocytochemistry using J2 antibody. Representative images of all conditions are shown. Scale bars, 50 and 10 μm for general plane and close-up panels respectively. **c** Levels of Alu-containing RNA as analysed by qRT-PCR using specific primers recognising Alu elements (n = 4). *p < 0.05 (Mann-Whitney *U*-test). **d** and **e** Markers of activated cellular reponse to dsRNA are upregulated in TDP-43 depleted cells. Levels of phosphorylated PKR and eIF2α were analysed by Western blot (**d**, representative blots are shown) and expression of *IFNB1* and an IFN-stimulated gene *CXCL10* - by qRT-PCR (**e**, n = 6). *p < 0.05 (Mann-Whitney *U*-test). **f** IFNbeta treatment stimulates NEAT1 expression and paraspeckle formation. NEAT1 levels were measured by qRT-PCR (n = 6). **p < 0.01 (Mann-Whitney *U*-test). Staining for an IFN-inducible protein IFIT3 was used as a positive control. Scale bar, 10 μm. **g** Simultaneous IFNbeta knockdown partially reverses the effect of TDP-43 depletion on paraspeckles. * and #p < 0.05, ***p < 0.001 (one-way ANOVA with Holm-Sidak correction for multiple comparisons). Scale bar, 10 μm. In all panels, cells were harvested for analysis 48 h post-transfection. Paraspeckles in panels **f** and **g** were visualised by NEAT1_2 RNA-FISH

We used J2 antibody, a gold standard for dsRNA detection [43], to study the presence of dsRNA species in cells depleted of TDP-43, Drosha, Dicer or ADAR1. An increase in J2-positive signal was obvious after knockdown of each of these genes (Fig. 4a and b, Additional file 3: Fig. S3a). In contrast, Ago2 or FUS knockdown did not cause dsRNA accumulation (Fig. 4b). J2 antibody was reported to recognise Alu repeats especially well [44]. We next used primers which specifically detect Alu-containing RNAs [45]. Dicer and Drosha but not TDP-43 or ADAR1 knockdown resulted in increased levels of Alu-containing RNAs as measured by qRT-PCR (Fig. 4c) indicating that a different repertoire of dsRNA species accumulate after knockdown of each gene.

The build-up of dsRNA is known to trigger phosphorylation of PKR and eIF2α and activation of type I interferon (IFN) signaling. In cells transfected with TDP-43 siRNA, levels of phosphorylated PKR and eIF2α were elevated (Fig. 4d; Additional file 3: Fig. S3b), and the expression of *IFNB1* and an IFN-stimulated gene (ISG) *CXCL10* was increased (Fig. 4e). Since dsRNA response eventually converges on type I IFNs, we asked whether these cytokines can contribute to paraspeckle response in TDP-43 depleted cells. Firstly, we showed that IFNbeta is the main type I IFN induced by dsRNA in neuroblastoma cells (Additional file 4: Fig. S4a). IFNbeta simulation per se was sufficient to stimulate paraspeckle assembly, although the effect was transient (Fig. 4f). In line with this, ligands of TLR3 (poly(I:C)) and TLR4 (bacterial lipopolysaccharide, LPS) which stimulate IFNbeta expression, but not a TLR2 ligand zymosan which does not affect IFNbeta production, were able to boost NEAT1 expression and paraspeckle assembly (Additional file 4: Fig. S4b-d). Finally, co-transfection of IFNbeta siRNA was sufficient to reduce paraspeckle abundance in cells transfected with TDP-43 siRNA although did not abrogate the hyper-assembly completely (Fig. 4g).

Taken together, these data suggest that endogenous dsRNA accumulation and associated type I IFN response represent one of the mechanisms behind augmented paraspeckle assembly caused by TDP-43 loss of function.

### Paraspeckles are protective in cells with compromised miRNA biogenesis and activated dsRNA response

**Fig. 5:**
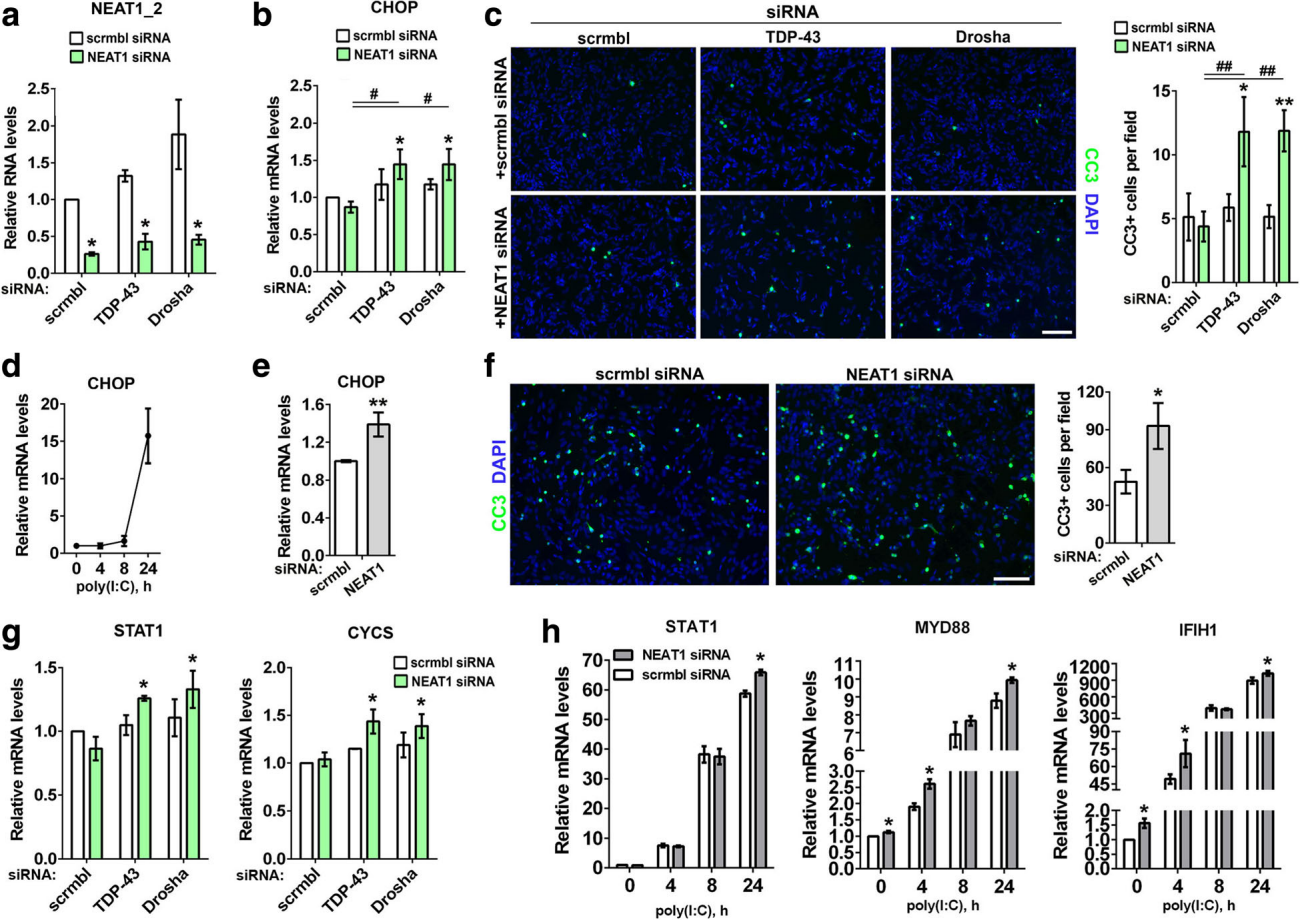
Loss of paraspeckles promotes apoptosis in cells with disturbed miRNA biogenesis and activated dsRNA response. **a-c** Disruption of paraspeckles in cells with downregulated TDP-43 or Drosha promotes apoptotic death in neuroblastoma cells. Efficiency of NEAT1_2 knockdown and levels of a proapototic protein CHOP mRNA were analysed by qRT-PCR (n = 3). * and #*p* ≤ 0.05 (Mann-Whitney *U*-test) (**a** and **b**). In **c**, representative images and quantification of cleaved caspase 3 (CC3) positive cells are shown. *p < 0.05, ** and ##p < 0.01 (one-way ANOVA). Scrambled siRNA or NEAT1 siRNA was co-transfected with an siRNA targeting TDP-43 or Drosha, and cells analysed 36 h post-transfection. * and # indicate statistically significant difference as compared to cells transfected with only scrambled siRNA or only NEAT1 siRNA, respectively. Scale bar, 100 μm. **d-f** Disruption of paraspeckles promotes apoptosis in dsRNA-stimulated cells. Cells were transfected with scrambled siRNA or NEAT1 siRNA and stimulated with poly(I:C) 36 h post-transfection. Induction of CHOP by poly(I:C) over time in normal cells (**d**) and CHOP mRNA levels in paraspeckle-deficient and paraspeckle-sufficient cells after 8 h of poly(I:C) stimulation (**e**) were analysed by qRT-PCR (n = 4). **p < 0.01 (Mann-Whitney *U*-test). In **f**, representative images and quantitation of CC3-positive cells in cultures transfected with scrambled siRNA or NEAT1 siRNA and treated with poly(I:C) for 24 h are shown. *p < 0.05 (Mann-Whitney *U*-test). Scale bar, 100 μm. **g** and **h** Expression of cytotoxicity-associated ISGs is potentiated by loss of paraspeckles in cells depleted of TDP-43 or Drosha (**g**) or stimulated by poly(I:C) (**h**). *N* = 3, *p < 0.05 (Mann-Whitney *U*-test)

We next examined whether paraspeckles confer protection to cells depleted of TDP-43 or stimulated with exogenous dsRNA. Neuroblastoma cells were transfected with siRNAs targeting TDP-43 or another Microprocessor component Drosha alone or in combination with NEAT1 siRNA. Cytotoxicity was assessed after 36 h using cleaved caspase 3 (CC3) and DNA damage-inducible transcript 3 also known as GADD153 or CHOP, a pro-apoptotic transcription factor [46, 47] as markers of apoptotic cell death. NEAT1 siRNA-mediated knockdown was equally efficient alone and in cells co-transfected with TDP-43 or Drosha siRNA, allowing 60% NEAT1_2 downregulation and loss of paraspeckles in the majority of cells (Fig. 5a and data not shown). We found that while knockdown of TDP-43 or Drosha alone did not result in significant cell death, simultaneous disruption of paraspeckles increased the rates of apoptosis for both genes studied (Fig. 5b and c). Although regulation of dsRNA response by paraspeckles is well documented [14], their ability to modulate dsRNA-induced apoptosis has not been addressed. We transfected cells with scrambled siRNA or NEAT1 siRNA and subsequently exposed them to a synthetic dsRNA analogue poly(I:C). Cultures depleted of paraspeckles had increased CHOP mRNA levels and higher numbers of CC3-positive cells after 8 h and 24 h of poly(I:C) stimulation, respectively (Fig. 5d-f).

How does paraspeckle deficiency promote apoptosis in cells with compromised miRNA biogenesis and activated dsRNA response? A whole class of cytotoxicity-associated IFN-stimulated genes (ISGs) were reported to be negatively regulated by miRNAs [48]. We found that potentially pro-apoptotic ISGs from this class, *STAT1* and *CYCS*, were significantly upregulated in paraspeckle-deficient cells depleted of TDP-43 and Drosha (Fig. 5g). Furthermore, three such ISGs, *STAT1, MYD88* and *IFIH1,* were consistently upregulated in NEAT1-depleted cells in the course of poly(I:C) stimulation (Fig. 5h).

Thus, paraspeckles are protective against apoptotic death in cells with compromised miRNA machinery and/or activated dsRNA response.

### Cultured human neurons lack paraspeckles but their de novo assembly can be triggered by dsRNA

**Fig. 6:**
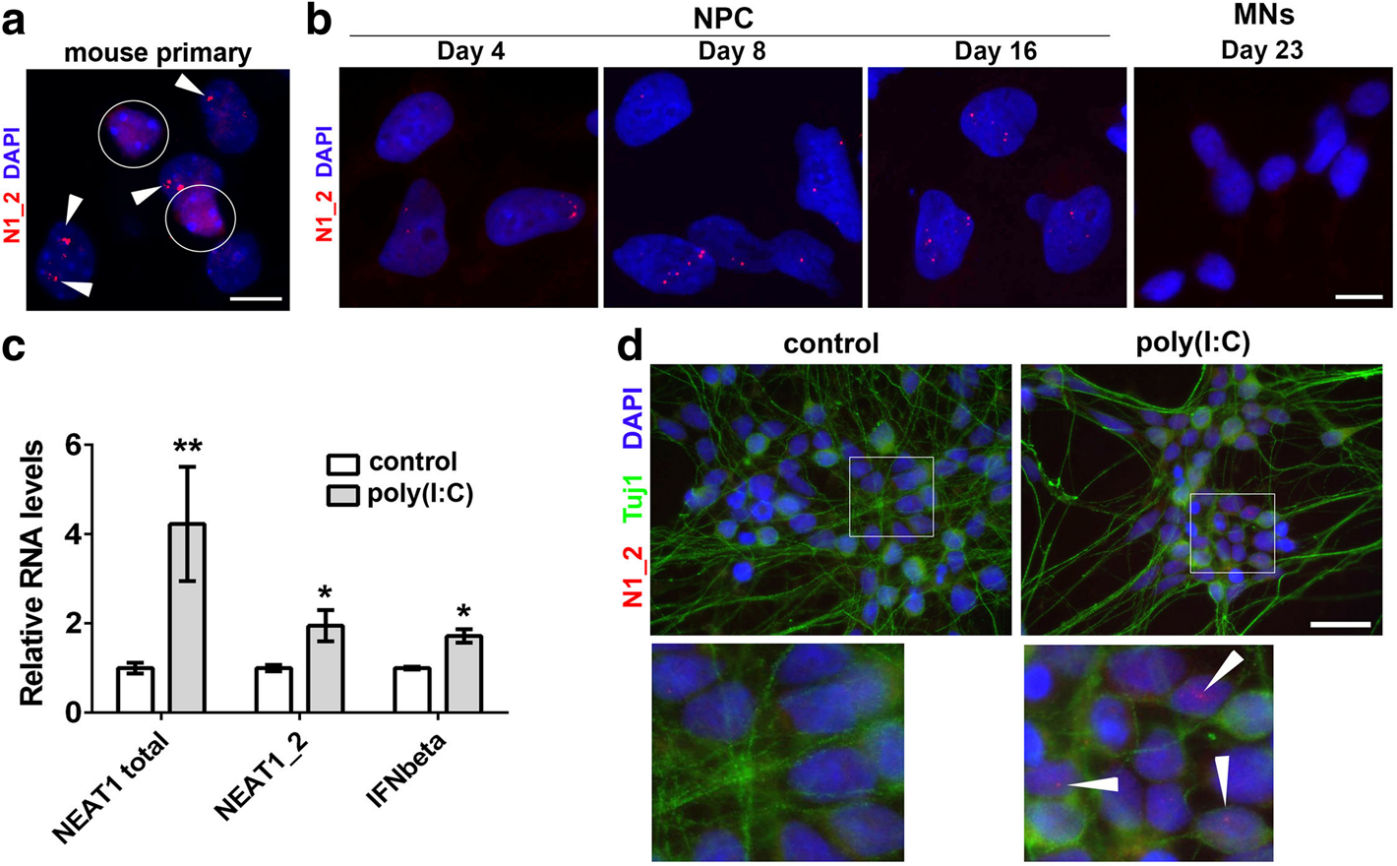
Post-mitotic neurons lack paraspeckles in vitro, but their assembly can be triggered by dsRNA. **a** Paraspeckles are present in glial cells but not in neurons in murine primary hippocampal cultures. Cultures were analysed at DIV14 by NEAT1_2 RNA-FISH. A representative image is shown. Neuronal nuclei are circled and paraspeckles in glial cells are indicated by arrowheads. **b** Paraspeckles are present in human neural precursor cells (NPCs) but disappear during their differentiation into motor neurons. Cultures were analysed at the indicated time-points by NEAT1_2 RNA-FISH. **c** and **d** Treatment of Day 40 cultures of human motor neurons with poly(I:C) leads to activation of IFN signaling, increased NEAT1 expression (**c**) and paraspeckle assembly in a fraction of cells (**d**, arrowheads). In **c**, gene expression was analysed by qRT-PCR (n = 4). *p < 0.05, **p < 0.01 (Mann-Whitney *U*-test). Scale bars, 10 μm in **a**, **b** and 20 μm in **d**

Normal postmitotic neurons in the brain or spinal cord of adult mice express very low levels of NEAT1_2 isoform and do not form paraspeckles in vivo [21]. However it was not clear whether neurons cultured in vitro would acquire and preserve paraspeckles. First, we studied primary hippocampal cultures from newborn mice. While glial cells have readily detectable paraspeckles already at 5 days in vitro, neurons had no sign of paraspeckles even after 14 days in vitro (Fig. 6a). Next we examined paraspeckle assembly during differentiation of human ES cells into motor neurons. Human ES cells lack paraspeckles but they appear during differentiation (days 4–5 into differentiation, trophoblast stage) [49]. Consistent with this study, we started observing paraspeckles in Day 4 neural precursor cells (NPCs), and they were prominent in Day 8 and Day 16 NPCs (Fig. 6b). However, paraspeckles were absent even in immature (Day 23) neurons and onwards (Fig. 6b). In contrast, retinoic acid/BDNF-induced differentiation of SH-SY5Y neuroblastoma cells for 6 days did not affect their ability to form paraspeckles (Additional file 5: Fig. S5), suggesting fundamental differences in the biology of neurons and neuroblastoma-derived neuron-like cells. Thus, disappearance of paraspeckles marks the transition between human NPCs and neurons, whereas de novo paraspeckle assembly is not triggered by in vitro conditions in primary neurons.

Finally, we tested whether inhibition of miRNA function, exposure to dsRNA or IFNbeta treatment can initiate paraspeckle formation in neurons. Human Day 40 motor neurons [30] were stimulated with poly(I:C) or treated with suramin or IFNbeta for 24 h. Induction of endogenous IFNbeta and NEAT1 was observed in poly(I:C)-treated neuronal cultures (Fig. 6c), and paraspeckles could be detected in a small fraction of poly(I:C)-stimulated neurons (Fig. 6d). In contrast, neither suramin nor IFNbeta induced paraspeckle assembly in neurons.

### Paraspeckle assembly can be promoted by a small molecule enhancer of miRNA biogenesis

Very few chemicals capable of stimulating paraspeckle assembly in cells with pre-existing paraspeckles have been identified so far. Proteasome inhibitors are known to promote NEAT1 synthesis and paraspeckle formation [13], however they are poor candidates as therapeutic molecules for ALS. Enoxacin is a small molecule enhancer of the miRNA pathway which stimulates the activity of the Dicer complex [50, 51]. In doing so, enoxacin increases levels of mature miRNAs thereby depleting miRNA precursors, pri-miRNA and pre-miRNA [51]. Recently, enoxacin been shown to ameliorate pathology in mouse models of ALS [52]. We studied the effect of enoxacin on paraspeckles in neuroblastoma cells. In addition, in our analysis we included HDAC inhibitors which were reported to stimulate NEAT1 expression [53] and two approved ALS therapeutics, riluzole and edaravone.

**Fig. 7:**
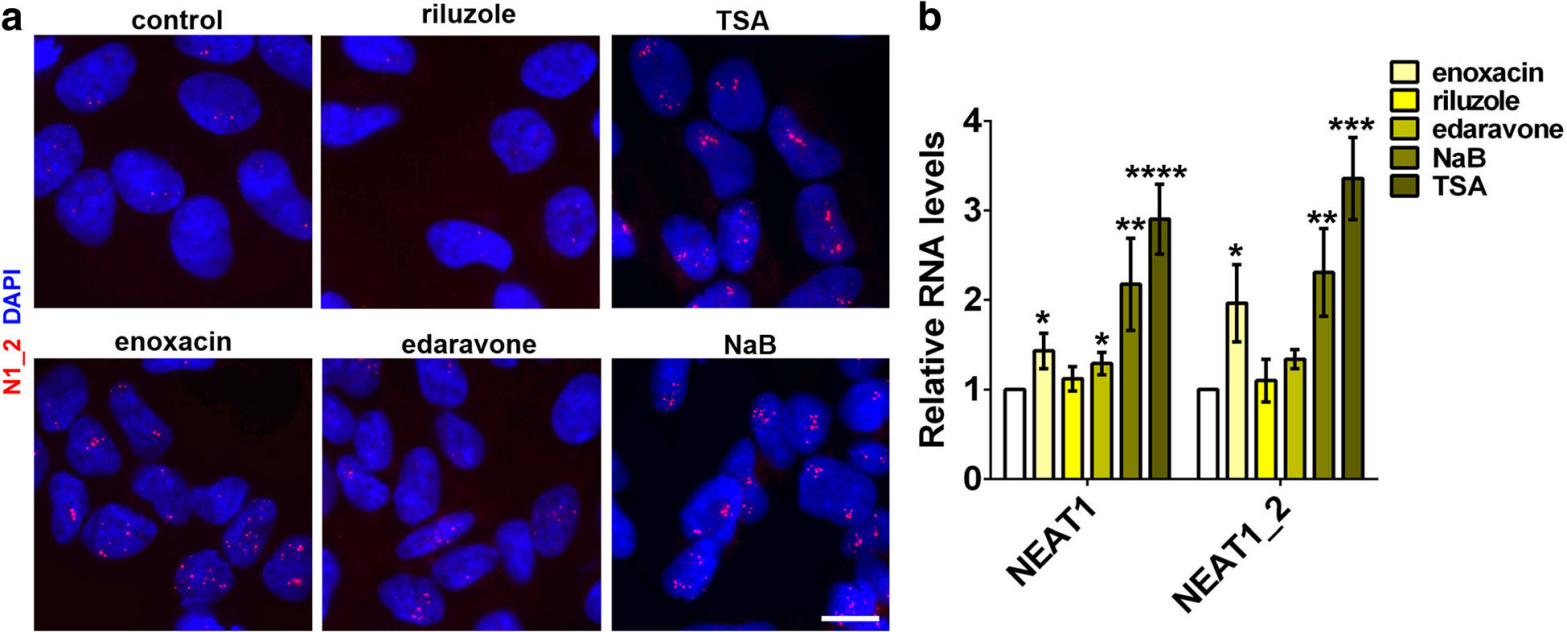
A small molecule enhancer of miRNA biogenesis stimulates paraspeckle assembly in neuroblastoma cells. **a** and **b** SH-SY5Y cells were treated with enoxacin, riluzole, edaravone and HDAC inhibitors trichostatin A (TSA) and sodium butyrate (NaB), and paraspeckle assembly was assessed by NEAT1_2 RNA-FISH (**a**) and qRT-PCR (n = 4–6, **b**). *p < 0.05, **p < 0.01, ***p < 0.001, ****p < 0.0001 (Kruskal-Wallis test with Dunn’s correction for multiple comparisons). Cells were harvested for analysis after 4 h of TSA and NaB treatment (500 nM and 2 mM, respectively) and after 24 h of enoxacin, riluzole and edaravone treatment (all 10 μM). Scale bar, 10 μm

Treatment with 10 μM enoxacin for 24 h increased NEAT1_2 levels and paraspeckle assembly in neuroblastoma cells (Fig. 7a and b); similar effect on paraspeckles was observed with short (4 h) treatment and with a higher enoxacin dose (Additional file 6: Fig. S6). Global HDAC inhibitors trichostatin A (TSA) and sodium butyrate (NaB) also significantly increased NEAT1_2 levels and led to the formation of large, elongated paraspeckles (Fig. 7a and b). Interestingly, edaravone but not riluzole also promoted paraspeckle biogenesis (Fig. 7a and b). None of the compounds studied was able to trigger de novo paraspeckles in hES cell derived motor neurons.

## Discussion

Nuclear bodies spatially organize and modulate various cellular processes [54]. Therefore it is not surprising that these membraneless organelles and their components have been implicated in multiple human diseases. Prominent examples are PML bodies and Gems linked to carcinogenesis and motor neuron degeneration, respectively [55, 56]. Paraspeckles have recently come into the limelight in the ALS field because of the extensive involvement of paraspeckle proteins in ALS pathogenesis. In the present study, we found that paraspeckle assembly in the spinal cord is shared by ALS cases with different aetiology and, as such, a hallmark of the disease. Using cell models, we identified two possible mechanisms which may initiate paraspeckle assembly in the spinal cord cells of the majority of ALS cases – compromised miRNA biogenesis and activated dsRNA response – both downstream of loss of TDP-43 function.

In the CNS, miRNAs are highly abundant and are subject to abnormal regulation in many neurodegenerative diseases, including ALS [57–61]. Levels of mature miRNAs in ALS spinal cord were reported to be globally reduced [52, 57, 62]. This dysregulation is consistent with TDP-43 loss of function in the majority of ALS cases since this protein is a known miRNA biogenesis factor [32]. An important role of paraspeckles in miRNA processing is supported by two recent studies [16, 63]. Thus, paraspeckle hyper-assembly in ALS motor neurons affected by TDP-43 loss of function may serve as one of the mechanisms to compensate for miRNA biogenesis deficiency. We also show that not only compromised function of the miRNA pathway but also its pharmacological enhancement results in paraspeckle hyper-assembly. This suggests that paraspeckles can respond to bi-directional changes in the activity of the miRNA pathway to either compensate for its compromised function or to meet the demand for miRNA precursors when its final step is over-active.

Another function of TDP-43 is acting as a chaperone to control RNA secondary structure [38] and therefore cellular dsRNA response. Paraspeckles are known to respond to exogenous dsRNA (viral and its analogues) [14], and here we show that abnormal accumulation of endogenous dsRNA can also initiate paraspeckle response. Given a significant crosstalk between miRNA and dsRNA response pathways [64], it is not surprising that paraspeckles function as a regulatory platform for both pathways. Although dsRNA response triggered by dysfunction of the miRNA pathway factors is mediated via different molecular sensors, including TLR3 (for Drosha, our unpublished observations), MyD88 (for Dicer) [65] and MDA5/RIG-I (for ADAR1) [66], it eventually converges on type I IFN. In contrast to the previous study [14], we found that IFNbeta treatment alone can stimulate NEAT1 expression and paraspeckle formation. This discrepancy is likely due to the transient effect of IFN treatment on paraspeckles which peaks at the 4-h time-point, whereas in the previous work, the 24 h time-point was examined. It is possible that IFN levels oscillate to maintain the dsRNA response active but at the same time preserve cellular viability [67]. Our in vitro data are consistent with a recent in vivo study demonstrating that TDP-43 knockdown in the adult murine nervous system leads to widespread upregulation of immune and, more specifically, antiviral genes [27]. Loss of TDP-43 function in the nervous system might be sufficient to trigger a chronic neuroinflammatory response.

In a previous study, siRNA-mediated TDP-43 knockdown led to decreased paraspeckle numbers in HeLa cells [8]. One possible explanation for this discrepancy is differences in cellular response to dsRNA and/or differences in the miRNA pathway regulation between the cell lines. Indeed, in the study on TDP-43 functions as an RNA chaperone, dsRNA was shown to be accumulated only in the nucleus of HeLa cells, whereas in neuroblastoma M17 cell line it was mainly cytoplasmic [38], similar to our study. Another possibility is the reliance of paraspeckle assembly on some TDP-43 function(s) specifically in HeLa cells.

Loss of TDP-43 function can explain paraspeckle hyper-assembly in the majority of ALS cases, i.e. almost all sALS cases as well as fALS cases caused by mutations in *TARDBP* and *C9ORF72* genes. Recently, Drosha has been identified as a component of C9orf72 dipeptide inclusions in patient’s neurons [68]. Therefore in fALS-C9 cases, loss of function for both TDP-43 and Drosha can jointly contribute to paraspeckle response. In a subset of sALS patients, activation of an endogenous retrovirus (ERV), HERV-K, was reported [69]. Elevated expression of ERVs can initiate dsRNA response [70, 71]. Activation of HERV-K may therefore contribute to paraspeckle hyper-assembly at least in some sALS cases. It still remains to be established whether paraspeckle formation is typical for other fALS cases such as those caused by mutations in genes encoding SOD1, FUS, TBK1 or OPTN and, if so, the underlying mechanisms. Many of ALS proteins function in miRNA and dsRNA metabolism, for example FUS is involved in miRNA biogenesis and miRNA-mediated silencing [35, 72], whereas TBK1 is one of the central factors in dsRNA response and type I IFN signaling. Thus compromised function of these pathways may represent a common mechanism behind paraspeckle response in different ALS cases.

Previously, paraspeckles were shown to be protective against cell death caused by proteasomal inhibition [13]. In current study, we show that paraspeckles confer protection to cells with compromised metabolism of miRNA and activated dsRNA response. Intriguingly, many of miRNA-controlled cytotoxicity-associated ISGs [48] were also reported to be regulated by paraspeckles either by sequestration of transcription factors or by nuclear retention of edited RNAs [14, 49, 73] suggesting a multi-layered control of cellular toxicity by paraspeckles. It should be noted however that the effect of paraspeckle disruption on survival in stable cell lines was small both in this and in the previous [13] report, despite the use of cells completely lacking NEAT1 and hence paraspeckles in the latter study. Such limited effect is in line with the fact that NEAT1 knockout mice do not have an overt phenotype [21] and further supports a modulatory role for paraspeckles in cellular responses (such as miRNA biogenesis, gene expression, RNA retention) which only becomes relevant under stressful/pathophysiological conditions. However, such modulatory activities of paraspeckles might be particularly important for neurons coping with neurodegeneration-inducing stresses.

## Conclusions

Paraspeckle hyper-assembly might be broadly neuroprotective in ALS. As a word of caution, however, these data were obtained in stable cell lines with pre-existing paraspeckles. Since normal post-mitotic neurons are free from paraspeckles, the impact of their de novo formation on neuronal metabolism may be much more dramatic than in stable cell lines. For example, it is coupled with changes in the levels of the short NEAT1 isoform with diverse paraspeckle-independent regulatory functions, including modulation of neuronal excitability [74–77]. Further studies, using neurons derived from human stem cells with ablated NEAT1_2 expression and from ALS patients’ iPS cells, are required to understand whether paraspeckles are protective for motor neurons in the disease context and in the long-term. This shall help us understand whether, how and when paraspeckles can be targeted for therapeutic purposes.

## Additional files

Additional file 1: **Figure S1.** The effect of TDP-43 dysfunction on paraspeckles, speckles and paraspeckle proteins in MCF7 and SH-SY5Y cells. a and b TDP-43 siRNA-mediated knockdown upregulates NEAT1_2 (a) and enhances paraspeckle assembly (b) in SH-SY5Y cells. Cells were transfected with scrambled siRNA or Silencer® TDP-43 siRNA and analysed by qRT-PCR (*n* = 6). Mean number of paraspeckles per cell was also quantified using NEAT1_2 RNA FISH. **p < 0.01, ***p < 0.001 (Mann-Whitney *U*-test in a and Student’s *t*-test in b). c and d Downregulation of TDP-43 using an esiRNA (endoribonuclease-prepared MISSION® esiRNA, c) or shRNA (d) stimulates paraspeckle assembly. The efficiency of knockdown was analysed by TDP-43 immunocytochemistry, and paraspeckle assembly – by NEAT1_2 RNA-FISH. Representative images are shown. Scale bars, 100 μm for left panels and 10 μm for right panels. e Speckles visualised by MALAT1 RNA-FISH are not affected by TDP-43 knockdown. Representative images are shown. Scale bar, 10 μm. f Levels of core paraspeckle proteins NONO, SFPQ and FUS are not affected by TDP-43 knockdown in SH-SY5Y cells. Representative Western blots are shown. g Sequences and positions of gRNAs used for disrupting the NLS of the endogenous TDP-43 protein by CRISPR/Cas9-mediated editing. Two combinations of upstream and downstream gRNA sequences within *TARDBP* gene selected to disrupt the NLS are shown. The sequence encoding for the NLS is given in blue and PAM sites are boxed. h Transient transfection of two combinations of plasmids encoding upstream and downstream gRNAs for targeting the NLS of TDP-43 results in partial redistribution of endogenous TDP-43 but does not lead to enhanced paraspeckle formation. Cells were analysed 72 h post-transfection. Representative images are shown, asterisks indicate cells with cytoplasmic TDP-43 redistribution. Scale bar, 10 μm. In a-f, cells were analysed 48 h post-transfection. (DOCX 517 kb)

Additional file 2: **Figure S2.** Drosha or TDP-43 downregulation affects miRNA processing. a Drosha knockdown leads to accumulation of miRNA precursors, pri-miR-17-92a and pri-miR-15a (*n* = 4–6). **p < 0.01 (one-way ANOVA with Holm-Sidak correction for multiple comparisons). Note the absence of significant accumulation of these pri-miRNAs in TDP-43 depleted cells, in accord with modulatory rather than essential function of this protein in miRNA processing in the nucleus. b Drosha or TDP-43 knockdown leads to downregulation of mature miRNAs processed from pri-miR-17-92a (*n* = 3). *p < 0.05; **p < 0.01 (one-way ANOVA with Holm-Sidak correction for multiple comparisons). Note that levels of all three mature miRNAs are significantly decreased in Drosha depleted cells, and TDP-43 knockdown also negatively affects two of the three miRNAs measured. (DOCX 157 kb)

Additional file 3: **Figure S3.** Accumulation of dsRNA (a) and increased levels of p-eIF2α (b) in MCF7 cells depleted of TDP-43. Cells were analysed 48 h post-transfection. Scale bars, 100 μm and 10 μm for general plane and close-up panels respectively. (DOCX 663 kb)

Additional file 4: **Figure S4.** The effect of IFN-inducing ligands on NEAT1 and paraspeckles. a IFNbeta is robustly induced by poly(I:C) in neuroblastoma cells. Cells were analysed by qRT-PCR after 24 h of poly(I:C) stimulation (*n* = 5). **p < 0.01 (Mann-Whitney *U*-test). b-d TLR3 and TLR4 ligands poly(I:C) and LPS, but not a TLR2 ligand zymosan, trigger IFNbeta response stimulating NEAT1 expression (b) and paraspeckle assembly (c). Cells were treated with poly(I:C), LPS or zymosan for 4 h and analysed by qRT-PCR (n = 3 or 4). *p < 0.05, **p < 0.01. NF-κB nuclear translocation was examined in parallel to confirm the activity of the compounds (d, asterisks indicate cells with nuclear NF-κB). Scale bar, 10 μm. (DOCX 333 kb)

Additional file 5: **Figure S5.** Differentiation of human neuroblastoma cells into neuron-like cells does not lead to the loss of paraspeckles. Differentiated SH-SY5Y cells develop extensive neurite network and are uniformly positive for a neuronal marker Tuj 1 (left panel) but preserve their ability to form paraspeckles (right panel). SH-SY5Y cells were induced to differentiate into neuron-like cells using retinoic acid/BDNF and analysed 6 days into differentiation by immunocytochemitry and NEAT1_2 RNA-FISH. Representative images are shown. Scale bars, 100 μm (left panel) and 10 μm (right panel). (DOCX 175 kb)

Additional file 6: **Figure S6.** Dose-dependent toxicity of enoxacin, edaravone and riluzole. a SH-SY5Y cells were treated with corresponding doses of compounds for 24 h, and toxicity was assessed using CellTiter Blue® Cell Viability Assay. *p < 0.05, **p < 0.01, ***p < 0.001, ****p < 0.0001 as compared to control (non-treated) cells (Kruskal-Wallis test with Dunn’s correction for multiple comparisons). b Enoxacin enhances paraspeckle assembly both with short (4 h) and prolonged (24 h) treatment at a non-toxic concentration of 50 μM. Representative images are shown. (DOCX 298 kb)

## Abbreviations

(F)ISH: (fluorescent) in situ hybridisation
ALS: Amyotrophic lateral sclerosis
dsRNA: double-stranded RNA
FTLD: Frontotemporal lobar degeneration
HDAC: Histone deacetylase
IFN: Interferon
ISG: Interferon-stimulated gene
LPS: Lipopolysaccharide
miRNA: microRNA
NEAT1: Nuclear enriched abundant transcript 1
NPC: Neural precursor cell
poly(I:C): Polyinosinic:polycytidylic acid
TDP-43: TAR DNA-binding protein 43
TSA: Trichostatin A

## References

[1] Renton AE, Chio A, Traynor BJ (2014). State of play in amyotrophic lateral sclerosis genetics. Nat Neurosci.

[2] DeJesus-Hernandez M, Mackenzie IR, Boeve BF, Boxer AL, Baker M, Rutherford NJ (2011). Expanded GGGGCC hexanucleotide repeat in noncoding region of C9ORF72 causes chromosome 9p-linked FTD and ALS. Neuron.

[3] Sreedharan J, Blair IP, Tripathi VB, Hu X, Vance C, Rogelj B (2008). TDP-43 mutations in familial and sporadic amyotrophic lateral sclerosis. Science.

[4] Lattante S, Rouleau GA, Kabashi E (2013). TARDBP and FUS mutations associated with amyotrophic lateral sclerosis: summary and update. Hum Mutat.

[5] Neumann M, Sampathu DM, Kwong LK, Truax AC, Micsenyi MC, Chou TT (2006). Ubiquitinated TDP-43 in frontotemporal lobar degeneration and amyotrophic lateral sclerosis. Science.

[6] Arai T, Hasegawa M, Akiyama H, Ikeda K, Nonaka T, Mori H (2006). TDP-43 is a component of ubiquitin-positive tau-negative inclusions in frontotemporal lobar degeneration and amyotrophic lateral sclerosis. Biochem Biophys Res Commun.

[7] Fox AH, Lamond AI (2010). Paraspeckles. Cold Spring Harb Perspect Biol.

[8] Naganuma T, Nakagawa S, Tanigawa A, Sasaki YF, Goshima N, Hirose T (2012). Alternative 3′-end processing of long noncoding RNA initiates construction of nuclear paraspeckles. EMBO J.

[9] West JA, Mito M, Kurosaka S, Takumi T, Tanegashima C, Chujo T, et al. Structural, super-resolution microscopy analysis of paraspeckle nuclear body organization. J Cell Biol. 2016; 10.1083/jcb.201601071. 10.1083/jcb.201601071 PMC5037409 27646274

[10] Clemson CM, Hutchinson JN, Sara SA, Ensminger AW, Fox AH, Chess A (2009). An architectural role for a nuclear noncoding RNA: NEAT1 RNA is essential for the structure of paraspeckles. Mol Cell.

[11] Hennig S, Kong G, Mannen T, Sadowska A, Kobelke S, Blythe A (2015). Prion-like domains in RNA binding proteins are essential for building subnuclear paraspeckles. J Cell Biol.

[12] Sunwoo H, Dinger ME, Wilusz JE, Amaral PP, Mattick JS (2009). Spector DL. MEN epsilon/beta nuclear-retained non-coding RNAs are up-regulated upon muscle differentiation and are essential components of paraspeckles. Genome Res.

[13] Hirose T, Virnicchi G, Tanigawa A, Naganuma T, Li R, Kimura H (2014). NEAT1 long noncoding RNA regulates transcription via protein sequestration within subnuclear bodies. Mol Biol Cell.

[14] Imamura K, Imamachi N, Akizuki G, Kumakura M, Kawaguchi A, Nagata K (2014). Long noncoding RNA NEAT1-dependent SFPQ relocation from promoter region to paraspeckle mediates IL8 expression upon immune stimuli. Mol Cell.

[15] Zhang Z, Carmichael GG (2001). The fate of dsRNA in the nucleus: a p54(nrb)-containing complex mediates the nuclear retention of promiscuously A-to-I edited RNAs. Cell.

[16] Jiang L, Shao C, Wu QJ, Chen G, Zhou J, Yang B (2017). NEAT1 scaffolds RNA-binding proteins and the microprocessor to globally enhance pri-miRNA processing. Nat Struct Mol Biol.

[17] Choudhry H, Albukhari A, Morotti M, Haider S, Moralli D, Smythies J (2015). Tumor hypoxia induces nuclear paraspeckle formation through HIF-2alpha dependent transcriptional activation of NEAT1 leading to cancer cell survival. Oncogene.

[18] Chakravarty D, Sboner A, Nair SS, Giannopoulou E, Li R, Hennig S (2014). The oestrogen receptor alpha-regulated lncRNA NEAT1 is a critical modulator of prostate cancer. Nat Commun.

[19] Adriaens C, Standaert L, Barra J, Latil M, Verfaillie A, Kalev P (2016). p53 induces formation of NEAT1 lncRNA-containing paraspeckles that modulate replication stress response and chemosensitivity. Nat Med.

[20] Nishimoto Y, Nakagawa S, Hirose T, Okano HJ, Takao M, Shibata S (2013). The long non-coding RNA nuclear-enriched abundant transcript 1_2 induces paraspeckle formation in the motor neuron during the early phase of amyotrophic lateral sclerosis. Molecular brain.

[21] Nakagawa S, Naganuma T, Shioi G, Hirose T (2011). Paraspeckles are subpopulation-specific nuclear bodies that are not essential in mice. J Cell Biol.

[22] Thomas-Jinu S, Gordon PM, Fielding T, Taylor R, Smith BN, Snowden V (2017). Non-nuclear pool of splicing factor SFPQ regulates axonal transcripts required for normal motor development. Neuron.

[23] Shelkovnikova TA, Robinson HK, Troakes C, Ninkina N, Buchman VL (2014). Compromised paraspeckle formation as a pathogenic factor in FUSopathies. Hum Mol Genet.

[24] Chesi A, Staahl BT, Jovicic A, Couthouis J, Fasolino M, Raphael AR (2013). Exome sequencing to identify de novo mutations in sporadic ALS trios. Nat Neurosci.

[25] Kim HJ, Kim NC, Wang YD, Scarborough EA, Moore J, Diaz Z (2013). Mutations in prion-like domains in hnRNPA2B1 and hnRNPA1 cause multisystem proteinopathy and ALS. Nature.

[26] Tollervey JR, Curk T, Rogelj B, Briese M, Cereda M, Kayikci M (2011). Characterizing the RNA targets and position-dependent splicing regulation by TDP-43. Nat Neurosci.

[27] Polymenidou M, Lagier-Tourenne C, Hutt KR, Huelga SC, Moran J, Liang TY (2011). Long pre-mRNA depletion and RNA missplicing contribute to neuronal vulnerability from loss of TDP-43. Nat Neurosci.

[28] Kukharsky MS, Quintiero A, Matsumoto T, Matsukawa K, An H, Hashimoto T (2015). Calcium-responsive transactivator (CREST) protein shares a set of structural and functional traits with other proteins associated with amyotrophic lateral sclerosis. Mol Neurodegener.

[29] Cong L, Ran FA, Cox D, Lin S, Barretto R, Habib N (2013). Multiplex genome engineering using CRISPR/Cas systems. Science.

[30] Shelkovnikova TA, Dimasi P, Kukharsky MS, An H, Quintiero A, Schirmer C (2017). Chronically stressed or stress-preconditioned neurons fail to maintain stress granule assembly. Cell Death Dis.

[31] Renton AE, Majounie E, Waite A, Simon-Sanchez J, Rollinson S, Gibbs JR (2011). A hexanucleotide repeat expansion in C9ORF72 is the cause of chromosome 9p21-linked ALS-FTD. Neuron.

[32] Kawahara Y, Mieda-Sato A (2012). TDP-43 promotes microRNA biogenesis as a component of the Drosha and dicer complexes. Proc Natl Acad Sci U S A.

[33] Buratti E, De Conti L, Stuani C, Romano M, Baralle M, Baralle F (2010). Nuclear factor TDP-43 can affect selected microRNA levels. FEBS J.

[34] Ota H, Sakurai M, Gupta R, Valente L, Wulff BE, Ariyoshi K (2013). ADAR1 forms a complex with dicer to promote microRNA processing and RNA-induced gene silencing. Cell.

[35] Morlando M, Dini Modigliani S, Torrelli G, Rosa A, Di Carlo V, Caffarelli E (2012). FUS stimulates microRNA biogenesis by facilitating co-transcriptional Drosha recruitment. EMBO J.

[36] Tan GS, Chiu CH, Garchow BG, Metzler D, Diamond SL, Kiriakidou M (2012). Small molecule inhibition of RISC loading. ACS Chem Biol.

[37] Lagier-Tourenne C, Polymenidou M, Hutt KR, Vu AQ, Baughn M, Huelga SC (2012). Divergent roles of ALS-linked proteins FUS/TLS and TDP-43 intersect in processing long pre-mRNAs. Nat Neurosci.

[38] Saldi TK, Ash PE, Wilson G, Gonzales P, Garrido-Lecca A, Roberts CM (2014). TDP-1, the Caenorhabditis elegans ortholog of TDP-43, limits the accumulation of double-stranded RNA. EMBO J.

[39] Ma H, Han P, Ye W, Chen H, Zheng X, Cheng L, et al. The long noncoding RNA NEAT1 exerts Antihantaviral effects by acting as positive feedback for RIG-I signaling. J Virol. 2017;91 10.1128/JVI.02250-16. 10.1128/JVI.02250-16 PMC5391460 28202761

[40] White E, Schlackow M, Kamieniarz-Gdula K, Proudfoot NJ, Gullerova M (2014). Human nuclear dicer restricts the deleterious accumulation of endogenous double-stranded RNA. Nat Struct Mol Biol.

[41] Heras SR, Macias S, Plass M, Fernandez N, Cano D, Eyras E (2013). The microprocessor controls the activity of mammalian retrotransposons. Nat Struct Mol Biol.

[42] Liddicoat BJ, Piskol R, Chalk AM, Ramaswami G, Higuchi M, Hartner JC (2015). RNA editing by ADAR1 prevents MDA5 sensing of endogenous dsRNA as nonself. Science.

[43] Weber F, Wagner V, Rasmussen SB, Hartmann R, Paludan SR (2006). Double-stranded RNA is produced by positive-strand RNA viruses and DNA viruses but not in detectable amounts by negative-strand RNA viruses. J Virol.

[44] Kaneko H, Dridi S, Tarallo V, Gelfand BD, Fowler BJ, Cho WG (2011). DICER1 deficit induces Alu RNA toxicity in age-related macular degeneration. Nature.

[45] Marullo M, Zuccato C, Mariotti C, Lahiri N, Tabrizi SJ, Di Donato S (2010). Expressed Alu repeats as a novel, reliable tool for normalization of real-time quantitative RT-PCR data. Genome Biol.

[46] Marciniak SJ, Yun CY, Oyadomari S, Novoa I, Zhang Y, Jungreis R (2004). CHOP induces death by promoting protein synthesis and oxidation in the stressed endoplasmic reticulum. Genes Dev.

[47] Matsumoto M, Minami M, Takeda K, Sakao Y, Akira S. Ectopic expression of CHOP (GADD153) induces apoptosis in M1 myeloblastic leukemia cells. FEBS Lett 1996; 395:143–147. doi: 0014–5793(96)01016–2. 10.1016/0014-5793(96)01016-2 8898082

[48] Seo GJ, Kincaid RP, Phanaksri T, Burke JM, Pare JM, Cox JE (2013). Reciprocal inhibition between intracellular antiviral signaling and the RNAi machinery in mammalian cells. Cell Host Microbe.

[49] Chen LL, Carmichael GG (2009). Altered nuclear retention of mRNAs containing inverted repeats in human embryonic stem cells: functional role of a nuclear noncoding RNA. Mol Cell.

[50] Melo S, Villanueva A, Moutinho C, Davalos V, Spizzo R, Ivan C (2011). Small molecule enoxacin is a cancer-specific growth inhibitor that acts by enhancing TAR RNA-binding protein 2-mediated microRNA processing. Proc Natl Acad Sci U S A.

[51] Shan G, Li Y, Zhang J, Li W, Szulwach KE, Duan R (2008). A small molecule enhances RNA interference and promotes microRNA processing. Nat Biotechnol.

[52] Emde A, Eitan C, Liou LL, Libby RT, Rivkin N, Magen I (2015). Dysregulated miRNA biogenesis downstream of cellular stress and ALS-causing mutations: a new mechanism for ALS. EMBO J.

[53] Schor IE, Lleres D, Risso GJ, Pawellek A, Ule J, Lamond AI (2012). Perturbation of chromatin structure globally affects localization and recruitment of splicing factors. PLoS One.

[54] Misteli T (2010). Higher-order genome organization in human disease. Cold Spring Harb Perspect Biol.

[55] de The H, Le Bras M, Lallemand-Breitenbach V. The cell biology of disease: acute promyelocytic leukemia, arsenic, and PML bodies. J Cell Biol 2012;198:11–21. doi: 10.1083/jcb.201112044. 10.1083/jcb.201112044 PMC3392943 22778276

[56] Yamazaki T, Chen S, Yu Y, Yan B, Haertlein TC, Carrasco MA (2012). FUS-SMN protein interactions link the motor neuron diseases ALS and SMA. Cell Rep.

[57] Figueroa-Romero C, Hur J, Lunn JS, Paez-Colasante X, Bender DE, Yung R (2016). Expression of microRNAs in human post-mortem amyotrophic lateral sclerosis spinal cords provides insight into disease mechanisms. Mol Cell Neurosci.

[58] Freischmidt A, Muller K, Ludolph AC, Weishaupt JH (2013). Systemic dysregulation of TDP-43 binding microRNAs in amyotrophic lateral sclerosis. Acta Neuropathol Commun.

[59] Gascon E, Gao FB (2014). The emerging roles of microRNAs in the pathogenesis of frontotemporal dementia-amyotrophic lateral sclerosis (FTD-ALS) spectrum disorders. J Neurogenet.

[60] Goodall EF, Heath PR, Bandmann O, Kirby J, Shaw PJ (2013). Neuronal dark matter: the emerging role of microRNAs in neurodegeneration. Front Cell Neurosci.

[61] Eitan C, Hornstein E (2016). Vulnerability of microRNA biogenesis in FTD-ALS. Brain Res.

[62] Campos-Melo D, Droppelmann CA, He Z, Volkening K, Strong MJ (2013). Altered microRNA expression profile in amyotrophic lateral sclerosis: a role in the regulation of NFL mRNA levels. Molecular brain..

[63] Bottini S, Hamouda-Tekaya N, Mategot R, Zaragosi LE, Audebert S, Pisano S (2017). Post-transcriptional gene silencing mediated by microRNAs is controlled by nucleoplasmic Sfpq. Nat Commun.

[64] Heyam A, Lagos D, Plevin M (2015). Dissecting the roles of TRBP and PACT in double-stranded RNA recognition and processing of noncoding RNAs. Wiley Interdiscip Rev RNA.

[65] Tarallo V, Hirano Y, Gelfand BD, Dridi S, Kerur N, Kim Y (2012). DICER1 loss and Alu RNA induce age-related macular degeneration via the NLRP3 inflammasome and MyD88. Cell.

[66] Mannion NM, Greenwood SM, Young R, Cox S, Brindle J, Read D (2014). The RNA-editing enzyme ADAR1 controls innate immune responses to RNA. Cell Rep.

[67] Ruggieri A, Dazert E, Metz P, Hofmann S, Bergeest JP, Mazur J (2012). Dynamic oscillation of translation and stress granule formation mark the cellular response to virus infection. Cell Host Microbe.

[68] Porta S, Kwong LK, Trojanowski JQ, Lee VM (2015). Drosha inclusions are new components of dipeptide-repeat protein aggregates in FTLD-TDP and ALS C9orf72 expansion cases. J Neuropathol Exp Neurol.

[69] Li W, Lee MH, Henderson L, Tyagi R, Bachani M, Steiner J (2015). Human endogenous retrovirus-K contributes to motor neuron disease. Sci Transl Med.

[70] Hurst TP, Magiorkinis G (2015). Activation of the innate immune response by endogenous retroviruses. J Gen Virol.

[71] Chiappinelli KB, Strissel PL, Desrichard A, Li H, Henke C, Akman B (2016). Inhibiting DNA methylation causes an interferon response in Cancer via dsRNA including endogenous retroviruses. Cell.

[72] Zhang T, Wu YC, Mullane P, Ji YJ, Liu H, He L, et al. FUS regulates activity of MicroRNA-mediated gene silencing. Mol Cell 2018; 69:787–801 e8. doi: 10.1016/j.molcel.2018.02.001. 10.1016/j.molcel.2018.02.001 PMC5836505 29499134

[73] Elbarbary RA, Li W, Tian B, Maquat LE (2013). STAU1 binding 3' UTR IRAlus complements nuclear retention to protect cells from PKR-mediated translational shutdown. Genes Dev.

[74] Barry G, Briggs JA, Hwang DW, Nayler SP, Fortuna PR, Jonkhout N (2017). The long non-coding RNA NEAT1 is responsive to neuronal activity and is associated with hyperexcitability states. Sci Rep.

[75] Li R, Harvey AR, Hodgetts SI, Fox AH. Functional dissection of NEAT1 using genome editing reveals substantial localisation of the NEAT1_1 isoform outside paraspeckles. RNA. 2017; 10.1261/rna.059477.116. 10.1261/rna.059477.116 PMC5435860 28325845

[76] Zhang F, Wu L, Qian J, Qu B, Xia S, La T (2016). Identification of the long noncoding RNA NEAT1 as a novel inflammatory regulator acting through MAPK pathway in human lupus. J Autoimmun.

[77] West JA, Davis CP, Sunwoo H, Simon MD, Sadreyev RI, Wang PI (2014). The long noncoding RNAs NEAT1 and MALAT1 bind active chromatin sites. Mol Cell.

