# Supplementary material for: Protective paraspeckle hyper-assembly downstream of TDP-43 loss of function in amyotrophic lateral sclerosis
Source: Mol Neurodegener. 2018 Jun 1;13:30. doi: 10.1186/s13024-018-0263-7 (PMC5984788; doi:10.1186/s13024-018-0263-7)
Supplement: Supplementary file 1 — Figure S1. The effect of TDP-43 dysfunction on paraspeckles, speckles and paraspeckle proteins in MCF7 and SH-SY5Y cells. a and b TDP-43 siRNA-mediated knockdown upregulates NEAT1_2 (a) and enhances paraspeckle assembly (b) in SH-SY5Y cells. Cells were transfected with scrambled siRNA or Silencer® TDP-43 siRNA and analysed by qRT-PCR (n = 6). Mean number of paraspeckles per cell was also quantified using NEAT1_2 RNA FISH. **p < 0.01, ***p < 0.001 (Mann-Whitney U-test in a and Student’s t-test in b). c and d Downregulation of TDP-43 using an esiRNA (endoribonuclease-prepared MISSION® esiRNA, c) or shRNA (d) stimulates paraspeckle assembly. The efficiency of knockdown was analysed by TDP-43 immunocytochemistry, and paraspeckle assembly – by NEAT1_2 RNA-FISH. Representative images are shown. Scale bars, 100 μm for left panels and 10 μm for right panels. e Speckles visualised by MALAT1 RNA-FISH are not affected by TDP-43 knockdown. Representative images are shown. Scale bar, 10 μm. f Levels of core paraspeckle proteins NONO, SFPQ and FUS are not affected by TDP-43 knockdown in SH-SY5Y cells. Representative Western blots are shown. g Sequences and positions of gRNAs used for disrupting the NLS of the endogenous TDP-43 protein by CRISPR/Cas9-mediated editing. Two combinations of upstream and downstream gRNA sequences within TARDBP gene selected to disrupt the NLS are shown. The sequence encoding for the NLS is given in blue and PAM sites are boxed. h Transient transfection of two combinations of plasmids encoding upstream and downstream gRNAs for targeting the NLS of TDP-43 results in partial redistribution of endogenous TDP-43 but does not lead to enhanced paraspeckle formation. Cells were analysed 72 h post-transfection. Representative images are shown, asterisks indicate cells with cytoplasmic TDP-43 redistribution. Scale bar, 10 μm. In a-f, cells were analysed 48 h post-transfection. (DOCX 517 kb) [file 13024_2018_263_MOESM1_ESM.docx]

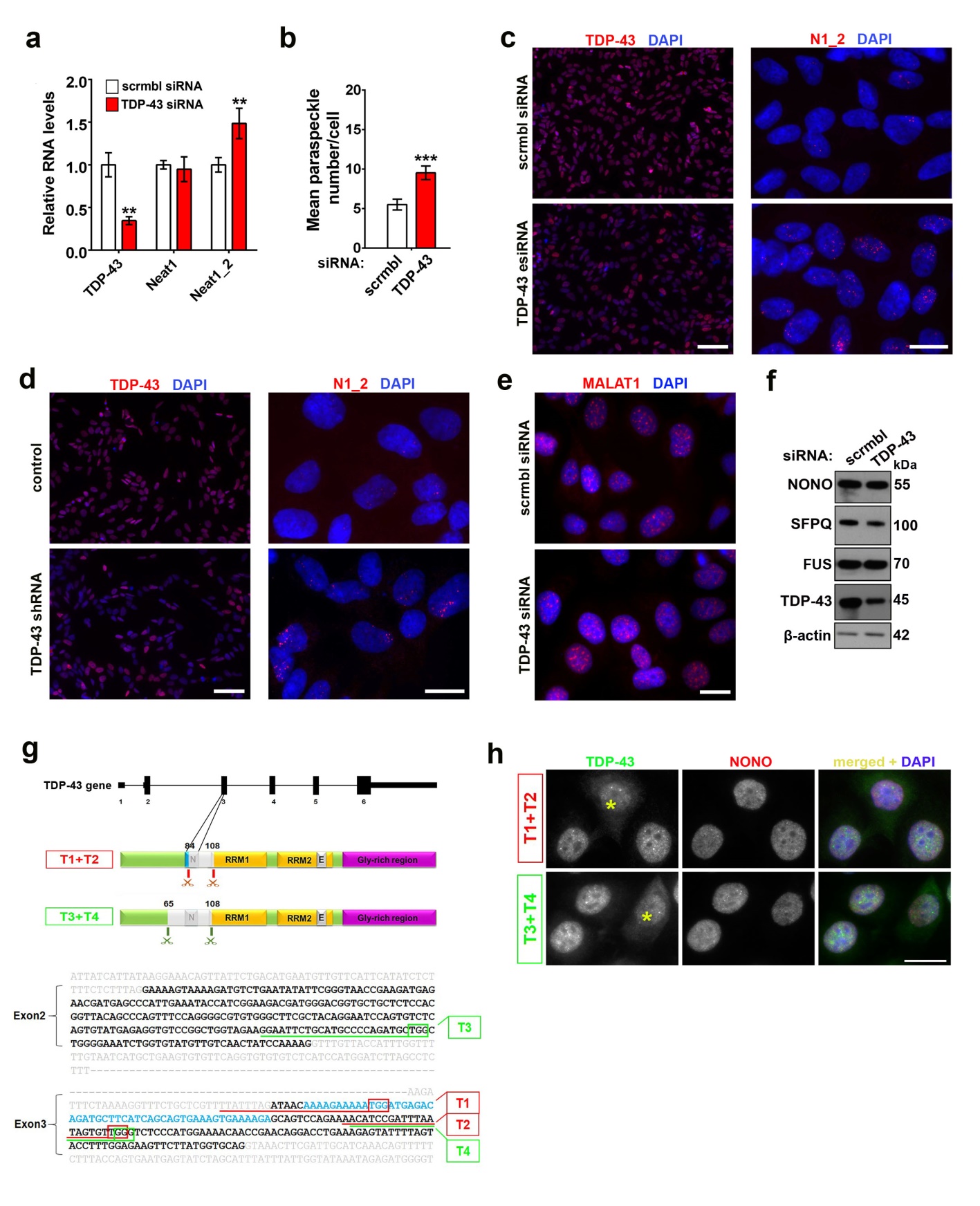


**Additional file 1: Figure S1. The effect of TDP-43 dysfunction on paraspeckles, speckles and paraspeckle proteins in MCF7 and SH-SY5Y cells.**

**a** and **b** TDP-43 siRNA-mediated knockdown upregulates NEAT1_2 (**a**) and enhances paraspeckle assembly (**b**) in SH-SY5Y cells. Cells were transfected with scrambled siRNA or Silencer® TDP-43 siRNA and analysed by qRT-PCR (n=6). Mean number of paraspeckles per cell was also quantified using NEAT1_2 RNA FISH. **p<0.01, ***p<0.001 (Mann-Whitney *U*-test in **a** and Student’s *t*-test in **b**).

**c** and **d** Downregulation of TDP-43 using an esiRNA (endoribonuclease-prepared MISSION® esiRNA, **c**) or shRNA (**d**) stimulates paraspeckle assembly. The efficiency of knockdown was analysed by TDP-43 immunocytochemistry, and paraspeckle assembly – by NEAT1_2 RNA-FISH. Representative images are shown. Scale bars, 100 µm for left panels and 10 µm for right panels.

**e** Speckles visualised by MALAT1 RNA-FISH are not affected by TDP-43 knockdown. Representative images are shown. Scale bar, 10 µm.

**f** Levels of core paraspeckle proteins NONO, SFPQ and FUS are not affected by TDP-43 knockdown in SH-SY5Y cells. Representative Western blots are shown.

**g** Sequences and positions of gRNAs used for disrupting the NLS of the endogenous TDP-43 protein by CRISPR/Cas9-mediated editing. Two combinations of upstream and downstream gRNA sequences within *TARDBP* gene selected to disrupt the NLS are shown. The sequence encoding for the NLS is given in blue and PAM sites are boxed.

**h** Transient transfection of two combinations of plasmids encoding upstream and downstream gRNAs for targeting the NLS of TDP-43 results in partial redistribution of endogenous TDP-43 but does not lead to enhanced paraspeckle formation. Cells were analysed 72 h post-transfection. Representative images are shown, asterisks indicate cells with cytoplasmic TDP-43 redistribution. Scale bar, 10 µm.

In **a-f**, cells were analysed 48 h post-transfection.
