# Supplementary material for: Protective paraspeckle hyper-assembly downstream of TDP-43 loss of function in amyotrophic lateral sclerosis
Source: Mol Neurodegener. 2018 Jun 1;13:30. doi: 10.1186/s13024-018-0263-7 (PMC5984788; doi:10.1186/s13024-018-0263-7)
Supplement: Supplementary file 2 — Figure S2. Drosha or TDP-43 downregulation affects miRNA processing. a Drosha knockdown leads to accumulation of miRNA precursors, pri-miR-17-92a and pri-miR-15a (n = 4–6). **p < 0.01 (one-way ANOVA with Holm-Sidak correction for multiple comparisons). Note the absence of significant accumulation of these pri-miRNAs in TDP-43 depleted cells, in accord with modulatory rather than essential function of this protein in miRNA processing in the nucleus. b Drosha or TDP-43 knockdown leads to downregulation of mature miRNAs processed from pri-miR-17-92a (n = 3). *p < 0.05; **p < 0.01 (one-way ANOVA with Holm-Sidak correction for multiple comparisons). Note that levels of all three mature miRNAs are significantly decreased in Drosha depleted cells, and TDP-43 knockdown also negatively affects two of the three miRNAs measured. (DOCX 157 kb) [file 13024_2018_263_MOESM2_ESM.docx]

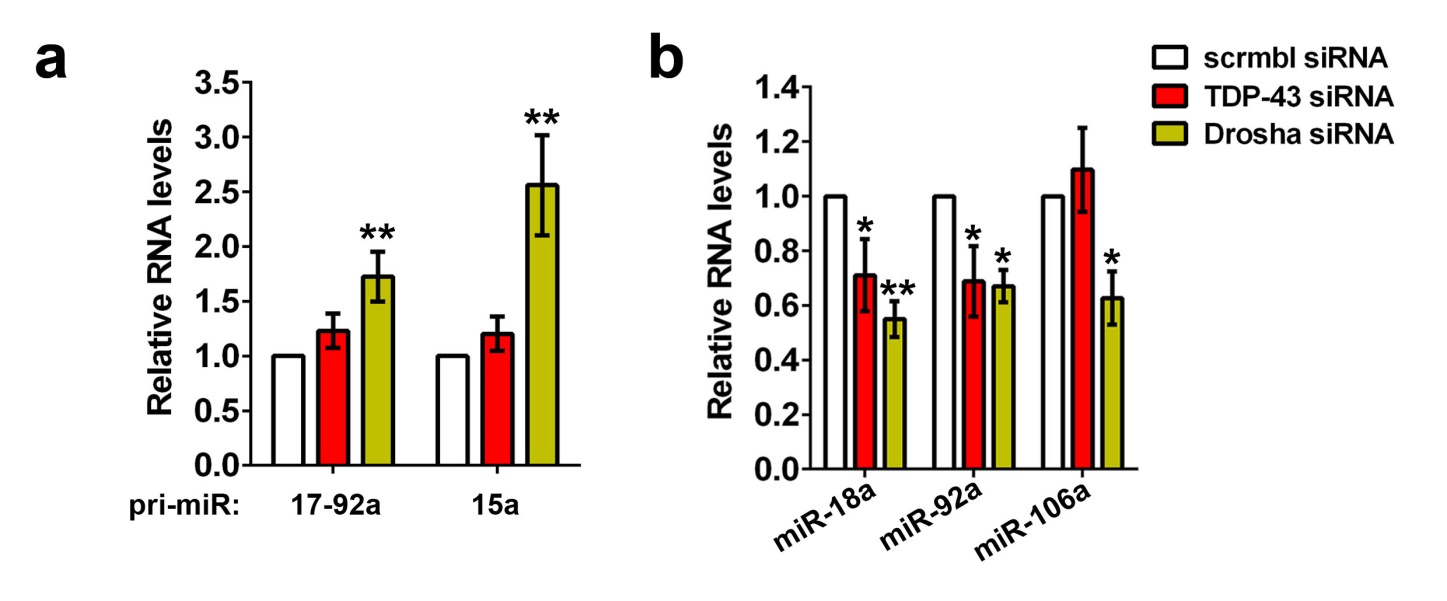


**Additional file 2: Figure S2.** **Drosha or TDP-43 downregulation affects miRNA processing.**

**a** Drosha knockdown leads to accumulation of miRNA precursors, pri-miR-17-92a and pri-miR-15a (n=4-6). **p<0.01 (one-way ANOVA with Holm-Sidak correction for multiple comparisons). Note the absence of significant accumulation of these pri-miRNAs in TDP-43 depleted cells, in accord with modulatory rather than essential function of this protein in miRNA processing in the nucleus.

**b** Drosha or TDP-43 knockdown leads to downregulation of mature miRNAs processed from pri-miR-17-92a (n=3). *p<0.05; **p<0.01 (one-way ANOVA with Holm-Sidak correction for multiple comparisons). Note that levels of all three mature miRNAs are significantly decreased in Drosha depleted cells, and TDP-43 knockdown also negatively affects two of the three miRNAs measured.
