# Supplementary material for: Protective paraspeckle hyper-assembly downstream of TDP-43 loss of function in amyotrophic lateral sclerosis
Source: Mol Neurodegener. 2018 Jun 1;13:30. doi: 10.1186/s13024-018-0263-7 (PMC5984788; doi:10.1186/s13024-018-0263-7)
Supplement: Supplementary file 3 — Figure S3. Accumulation of dsRNA (a) and increased levels of p-eIF2α (b) in MCF7 cells depleted of TDP-43. Cells were analysed 48 h post-transfection. Scale bars, 100 μm and 10 μm for general plane and close-up panels respectively. (DOCX 663 kb) [file 13024_2018_263_MOESM3_ESM.docx]

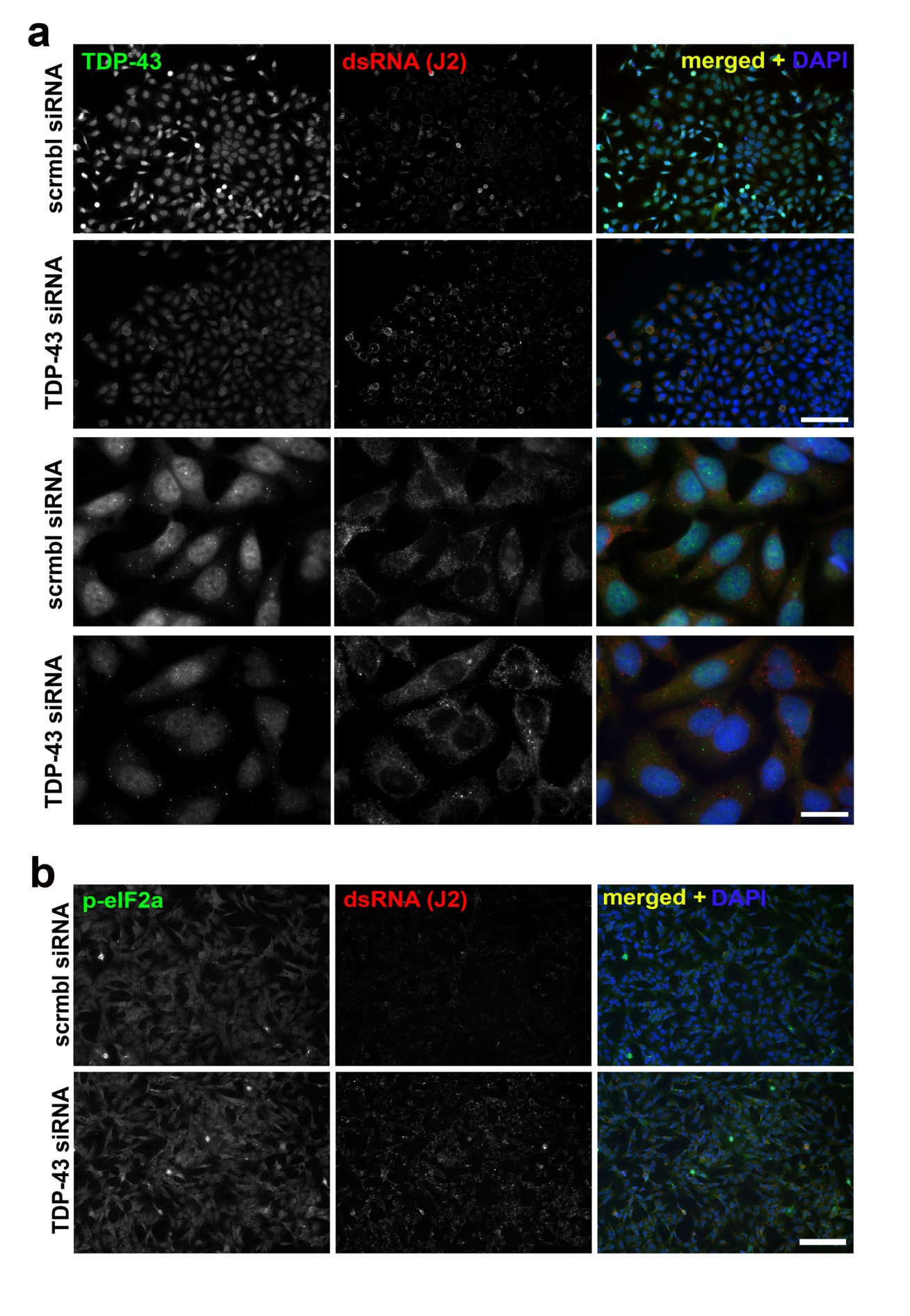


**Additional file 3: Figure S3. Accumulation of dsRNA (a) and increased levels of p-eIF2α (b) in MCF7 cells depleted of TDP-43.**

Cells were analysed 48 h post-transfection. Scale bars, 100 µm and 10 µm for general plane and close-up panels respectively.
