# Supplementary material for: Protective paraspeckle hyper-assembly downstream of TDP-43 loss of function in amyotrophic lateral sclerosis
Source: Mol Neurodegener. 2018 Jun 1;13:30. doi: 10.1186/s13024-018-0263-7 (PMC5984788; doi:10.1186/s13024-018-0263-7)
Supplement: Supplementary file 4 — Figure S4. The effect of IFN-inducing ligands on NEAT1 and paraspeckles. a IFNbeta is robustly induced by poly(I:C) in neuroblastoma cells. Cells were analysed by qRT-PCR after 24 h of poly(I:C) stimulation (n = 5). **p < 0.01 (Mann-Whitney U-test). b-d TLR3 and TLR4 ligands poly(I:C) and LPS, but not a TLR2 ligand zymosan, trigger IFNbeta response stimulating NEAT1 expression (b) and paraspeckle assembly (c). Cells were treated with poly(I:C), LPS or zymosan for 4 h and analysed by qRT-PCR (n = 3 or 4). *p < 0.05, **p < 0.01. NF-κB nuclear translocation was examined in parallel to confirm the activity of the compounds (d, asterisks indicate cells with nuclear NF-κB). Scale bar, 10 μm. (DOCX 333 kb) [file 13024_2018_263_MOESM4_ESM.docx]

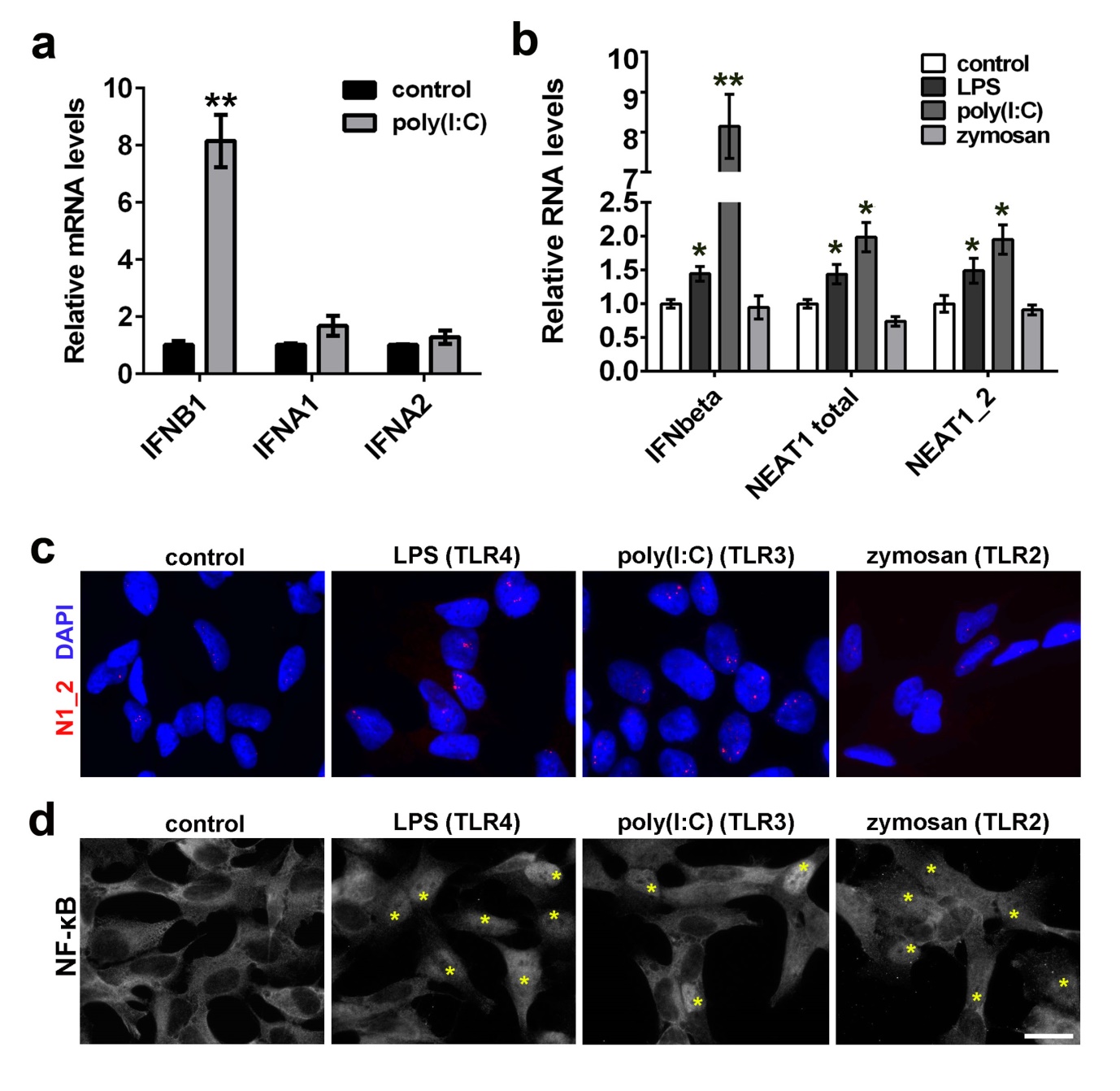


**Additional file 4: Figure S4. The effect of IFN-inducing ligands on NEAT1 and paraspeckles.**

**a** IFNbeta is robustly induced by poly(I:C) in neuroblastoma cells. Cells were analysed by qRT-PCR after 24 h of poly(I:C) stimulation (n=5). **p<0.01 (Mann-Whitney *U*-test).

**b-d** TLR3 and TLR4 ligands poly(I:C) and LPS, but not a TLR2 ligand zymosan, trigger IFNbeta response stimulating NEAT1 expression (**b**) and paraspeckle assembly (**c**). Cells were treated with poly(I:C), LPS or zymosan for 4 h and analysed by qRT-PCR (n=3 or 4). *p<0.05, **p<0.01. NF-κB nuclear translocation was examined in parallel to confirm the activity of the compounds (**d**, asterisks indicate cells with nuclear NF-κB). Scale bar, 10 µm.
