# Supplementary material for: Protective paraspeckle hyper-assembly downstream of TDP-43 loss of function in amyotrophic lateral sclerosis
Source: Mol Neurodegener. 2018 Jun 1;13:30. doi: 10.1186/s13024-018-0263-7 (PMC5984788; doi:10.1186/s13024-018-0263-7)
Supplement: Supplementary file 5 — Figure S5. Differentiation of human neuroblastoma cells into neuron-like cells does not lead to the loss of paraspeckles. Differentiated SH-SY5Y cells develop extensive neurite network and are uniformly positive for a neuronal marker Tuj 1 (left panel) but preserve their ability to form paraspeckles (right panel). SH-SY5Y cells were induced to differentiate into neuron-like cells using retinoic acid/BDNF and analysed 6 days into differentiation by immunocytochemitry and NEAT1_2 RNA-FISH. Representative images are shown. Scale bars, 100 μm (left panel) and 10 μm (right panel). (DOCX 175 kb) [file 13024_2018_263_MOESM5_ESM.docx]

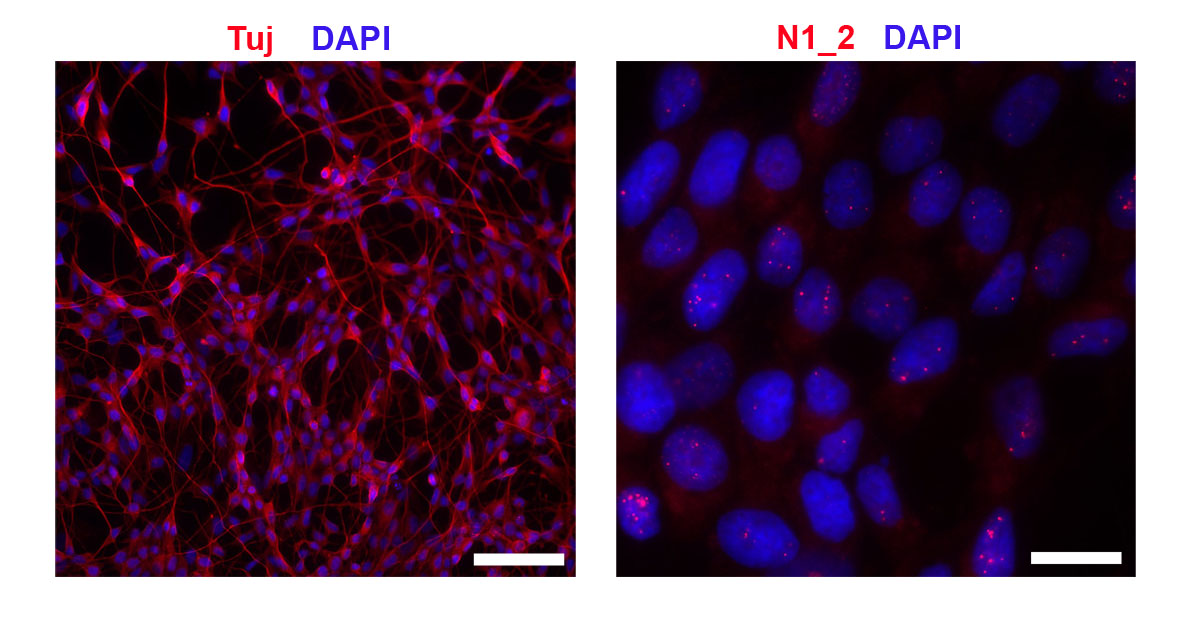


**Additional file 5: Figure S5. Differentiation of human neuroblastoma cells into neuron-like cells does not lead to the loss of paraspeckles.**

Differentiated SH-SY5Y cells develop extensive neurite network and are uniformly positive for a neuronal marker Tuj 1 (left panel) but preserve their ability to form paraspeckles (right panel). SH-SY5Y cells were induced to differentiate into neuron-like cells using retinoic acid/BDNF and analysed 6 days into differentiation by immunocytochemitry and NEAT1_2 RNA-FISH. Representative images are shown. Scale bars, 100 µm (left panel) and 10 µm (right panel).
