# Supplementary material for: Protective paraspeckle hyper-assembly downstream of TDP-43 loss of function in amyotrophic lateral sclerosis
Source: Mol Neurodegener. 2018 Jun 1;13:30. doi: 10.1186/s13024-018-0263-7 (PMC5984788; doi:10.1186/s13024-018-0263-7)
Supplement: Supplementary file 6 — Figure S6. Dose-dependent toxicity of enoxacin, edaravone and riluzole. a SH-SY5Y cells were treated with corresponding doses of compounds for 24 h, and toxicity was assessed using CellTiter Blue® Cell Viability Assay. *p < 0.05, **p < 0.01, ***p < 0.001, ****p < 0.0001 as compared to control (non-treated) cells (Kruskal-Wallis test with Dunn’s correction for multiple comparisons). b Enoxacin enhances paraspeckle assembly both with short (4 h) and prolonged (24 h) treatment at a non-toxic concentration of 50 μM. Representative images are shown. (DOCX 298 kb) [file 13024_2018_263_MOESM6_ESM.docx]

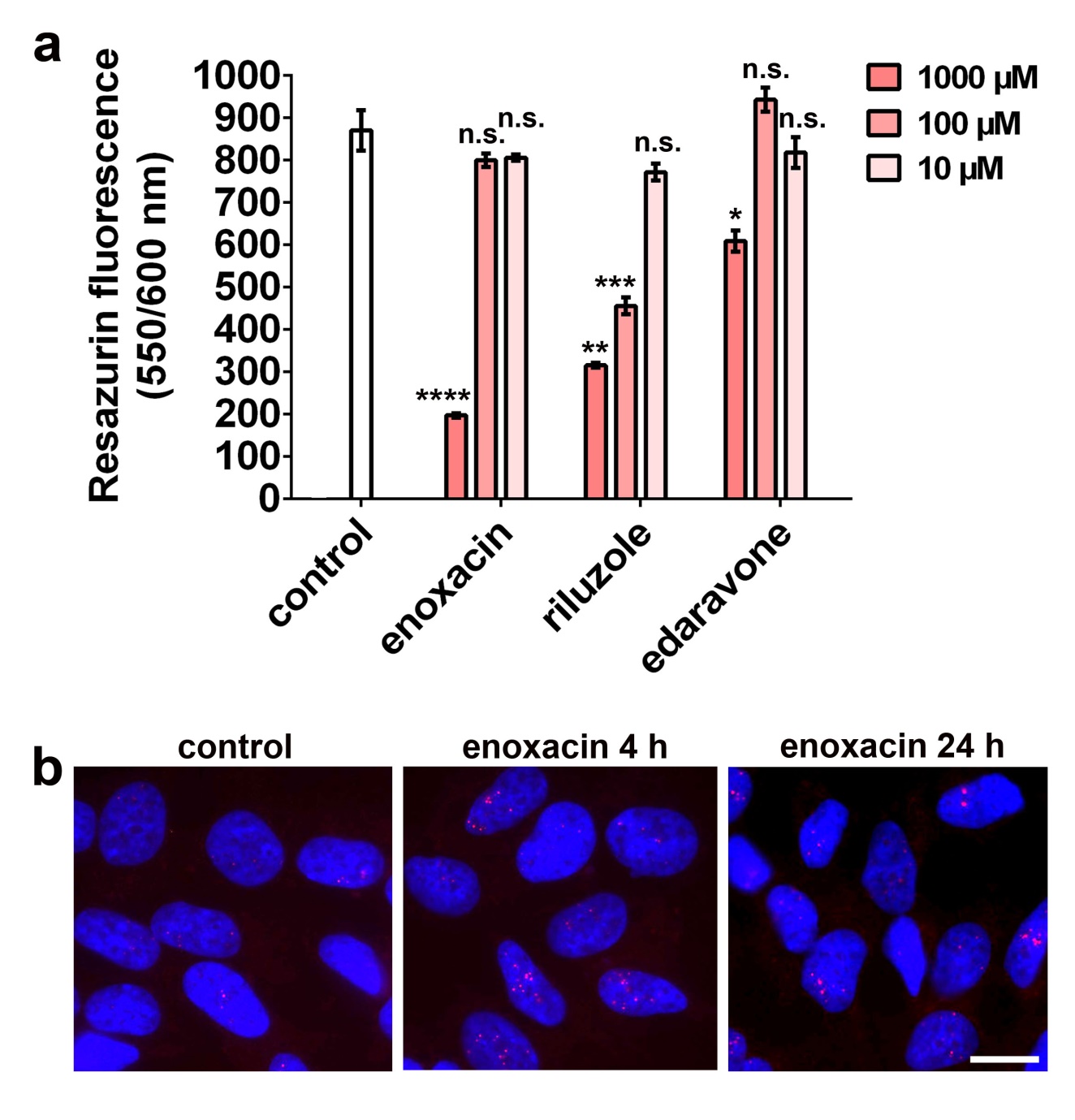


**Additional file 6: Figure S6. Dose-dependent toxicity of enoxacin, edaravone and riluzole.**

**a** SH-SY5Y cells were treated with corresponding doses of compounds for 24 hours, and toxicity was assessed using CellTiter Blue® Cell Viability Assay. *p<0.05, **p<0.01, ***p<0.001, ****p<0.0001 as compared to control (non-treated) cells (Kruskal-Wallis test with Dunn’s correction for multiple comparisons).

**b** Enoxacin enhances paraspeckle assembly both with short (4 h) and prolonged (24 h) treatment at a non-toxic concentration of 50 µM. Representative images are shown.
